# Identification and Expression Analyses of Invertase Genes in Moso Bamboo Reveal Their Potential Drought Stress Functions

**DOI:** 10.3389/fgene.2021.696300

**Published:** 2021-08-30

**Authors:** Chenglei Zhu, Kebin Yang, Guangzhu Li, Ying Li, Zhimin Gao

**Affiliations:** ^1^Institute of Gene Science and Industrialization for Bamboo and Rattan Resources, International Center for Bamboo and Rattan, Beijing, China; ^2^Key Laboratory of National Forestry and Grassland Administration, Beijing for Bamboo and Rattan Science and Technology, Beijing, China

**Keywords:** *Phyllostachys edulis*, invertase, gene identification, expression pattern, drought stress

## Abstract

Invertases (INVs) can irreversibly hydrolyze sucrose into fructose and glucose, which play principal roles in carbon metabolism and responses to various stresses in plants. However, little is known about the INV family in bamboos, especially their potential function in drought stress. In this study, 29 *PeINV*s were identified in moso bamboo (*Phyllostachys edulis*). They were clustered into alkaline/neutral invertase (NINV) and acid invertase (AINV) groups based on the gene structures, conserved motifs, and phylogenetic analysis results. The collinearity analysis showed nine segmental duplication pairs within *PeINV*s, and 25 pairs were detected between *PeINV*s and *OsINV*s. *PeINV*s may have undergone strong purification selection during evolution, and a variety of stress and phytohormone-related regulatory elements were found in the promoters of *PeINV*s. The tissue-specific expression analysis showed that *PeINV*s were differentially expressed in various moso bamboo tissues, which suggested that they showed functional diversity. Both the RNA-seq and quantitative real-time PCR results indicated that four *PeINV*s were significantly upregulated under drought stress. Co-expression network and Pearson’s correlation coefficient analyses showed that these *PeINV*s co-expressed positively with sugar and water transport genes (*SWTG*s), and the changes were consistent with sugar content. Overall, we speculate that the identified *PeINV*s are spatiotemporally expressed, which enables them to participate in moso bamboo growth and development. Furthermore, *PeINV*s, together with *SWTG*s, also seem to play vital roles in the response to drought stress. These results provide a comprehensive information resource for *PeINV*s, which will facilitate further study of the molecular mechanism underlying *PeINV*s involvement in the response to drought stress in moso bamboo.

## Introduction

Carbon autotrophy is a prominent characteristic of higher plants (Roitsch and González, 2004). Sucrose is the main carbohydrate produced by photosynthetically active tissues and is translocated through the phloem from the source leaves to sink organs as a transport molecule (Tiessen and Padilla-Chacon, 2012; Bihmidine et al., 2013). The absorption of sucrose from sinks can occur either directly or through the cleavage of sucrose (Cho et al., 2005). Sucrose can be reversibly hydrolyzed to UDP-glucose and fructose by sucrose synthase (SuSy; EC 2.4.1.13) (Koch, 2004) and irreversibly divided into simple hexoses (glucose and fructose) by invertases (INV; EC 3.2.1.26) (Tang et al., 1999). The present results show that SuSy mainly participates in the biosynthesis of sugar polymers (Roitsch and González, 2004; Hummel et al., 2009). In addition, INV is known to be widely involved in regulating plant growth and development, such as reproductive development and stress tolerance responses (Koch, 2004; Barratt et al., 2009; Wang and Ruan, 2016). In particular, sucrose and its hydrolysis products are indispensable in gene expression, signal transduction, and stress responses to plant growth (Ruan, 2014).

The INVs of higher plants are classified into two subfamilies based on their optimal pH: acid invertases (AINVs) (pH between 4.5 and 5.0) and alkaline/neutral invertases (NINVs) (pH 6.5 to 8.0) (Salerno and Curatti, 2003), respectively. NINVs are believed to be closely related to cyanobacterial invertases, which belong to glycoside hydrolase family 100 (GH 100) (Vargas et al., 2003). In contrast, AINVs arise from respiratory eukaryotes and aerobic bacteria, which belong to glycoside hydrolase family 32 (GH 32) (Sturm and Chrispeels, 1990) and are classified as cell-wall invertases (CWINVs) and vacuole invertases (VINVs) according to their location in cell wall and vacuole, respectively. Although CWINVs and VINVs are found in different locations, they share some common biochemical properties (Ruan et al., 2010). For example, they both catalyze sucrose and other β-fructoses, including oligosaccharides, therefore, they are also referred to as β-fructofuranosidases (Stitt et al., 1991). In contrast, NINVs, which lack the N-terminal signal peptide and are not β-fructofuranosidases, specifically catalyze sucrose (Sturm, 1999). There are two conserved motifs, which are the β-fructosidase motif (NDPD/NG) and the cysteine catalytic domain motif (WECV/PD). They are associated with AINVs active site (Verhaest et al., 2006; Canam et al., 2008). Little is known about NINV functions and their regulatory mechanisms because it is difficult to purify them and determine their expressions (Koch, 2004).

In the past decades, with the development of genome sequencing technology, INV family genes have been identified in many plants, such as *Arabidopsis thaliana*, *Oryza sativa* (Ji et al., 2005), *Triticum aestivum* (Francki et al., 2006), *Zea mays* (Koch et al., 1996), *Populus trichocarpa* (Chen et al., 2015), and *Capsicum annuum* (Shen et al., 2018a, b). The functional characterization of INVs and their regulatory mechanisms have also been widely documented. Many studies on various plants have confirmed that *CWINV*s play essential roles in the regulation of sucrose partitioning (Koch et al., 1996), seed and pollen development (Chourey et al., 2006), and environmental responses (Proels and Hückelhoven, 2014). Furthermore, VINVs have been found to function in fruits and storage organs (Yu et al., 2008), and during drought and hypoxia stress (Roitsch and González, 2004). *Incw2* mRNA levels increased in the ovary after pollination and tended to be lower in response to drought stress (Andersen et al., 2002). *IVR2* plays different regulatory roles in the sink and source organs of maize under drought stress (Kim et al., 2000), and *OsVIN2* is upregulated rather than downregulated in rice under drought stress (Ji et al., 2005). The proposed functions of NINVs have also been gradually identified, including sugar signal transduction (Lou et al., 2007), cellulose biosynthesis (Rende et al., 2017), shoot and root growth (Martín et al., 2013), flower and fruit development (Roitsch and González, 2004), and response to biological and abiotic stresses (Liu et al., 2015). *A. thaliana* plants that overexpress *PtrA/NINV* have a better capacity to adjust their osmotic potential in response to drought stress (Dahro et al., 2016). The function of INVs is diverse in plants, and a comprehensive analysis of INVs may improve our understanding of their function during in the sucrose metabolism in plants.

Moso bamboo (*Phyllostachys edulis*), which belongs to the Bambusoideae of the Poaceae, and is one of the most important bamboo species in China, occupies a forestland area of 4.68 million hm^2^, accounting for 72.96% of the bamboo forest in China (Li and Feng, 2019). Moso bamboo can grow more than 1 m in height per day during its rapid growth period and its height growth within one and a half months (Peng et al., 2013). It has been reported that *PeAINV*s may be involved in internode elongation in moso bamboo shoots (Guo et al., 2020). However, drought is one of the leading detrimental environmental factors affecting the rapid growth of moso bamboo. It has been reported that the transcription factors (bHLH and WRKY) and structural genes (*LEA*, *UGE*, *ZEP*, and *AQP*) participate in the response to drought stress (Huang et al., 2016b; Sun et al., 2016; Li et al., 2017; Lou et al., 2017; Sun et al., 2017; Cheng et al., 2018). Furthermore, sugar transport pathway genes also play essential roles in the response to stress (Roitsch and González, 2004; Ruan et al., 2010). However, the possible role of INVs in bamboo remains unclear. Understanding the molecular characteristics and evolution of INV family members is the first step in studying their function. In this study, a comprehensive analysis of INV gene members was conducted based on the updated genome for moso bamboo (Zhao et al., 2018). It mainly focused on the gene structure, conserved motifs, sequence phylogeny, gene syntenic, expression patterns in various tissues and in leaves of moso bamboo under drought stress. We also built a co-expression network for *PeINV*s, sugar and water transport genes (*SWTG*s), whose expression patterns were further validated by qRT-PCR. This study aimed to: (1) identify the *INV*s members in moso bamboo; (2) analyze the genetic diversity and functional differentiation of *PeINV*s; and (3) validate the expression patterns and relationships of *PeINV*s and *SWTG*s under drought stress.

## Materials and Methods

### Identification of Invertase Genes in Moso Bamboo

We used two different approaches to annotate and identify the *INV* genes in moso bamboo. First, the known sequences of *INV*s from the *O. sativa* and *A. thaliana* databases (Supplementary Table 1) were used as queries to search for potential *INV*s in the moso bamboo genome database^1^ using the Basic Local Alignment Tool for Protein (BLASTP 2.9.0) program (*e*-value < 1 × 10^–5^). Second, HMMRE 3.0 software was used to identify *INV*s using hidden Markov model (HMM) profile of the invertase domain (NINV: PF12899, CWINV: PF08244 and PF00251, and VINV: PF11837, PF08244, and PF00251) from the Pfam database as queries (*e*-value < 1 × 10^–5^). Subsequently, each putative INV was further examined for the presence of conserved domains by submitting them to Pfam^2^ (Finn et al., 2014) and the simple modular architecture research tool (SMART) database^3^ (Letunic et al., 2012), and the sequences with complete conserved domains were preserved. In addition, the *INV*s in *Bonia amplexicaulis* and *Olyra latifolia* were also investigated and used for comparative analysis.

### Sequence Analysis, Protein Properties, and Phylogenetic Tree Construction of Invertase Family Members

A gene structure analysis of *INV*s was performed using the Gene Structure Display Server^4^ (Hu et al., 2015). The ExPASy website (ProtParam^5^) was used to calculate the molecular weight, theoretical isoelectric point (pI), and protein instability index of predicted INV proteins in three bamboo species (Gasteiger et al., 2003). MEME server^6^ and TBtools 1.08 were employed to analyze the motif composition of INV proteins in three bamboo species (Brown et al., 2013; Chen et al., 2020).

The amino acid sequences of NINVs and AINVs from six plant species (*P. edulis*, *B. amplexicaulis*, *O. latifolia, O. sativa*, *A. thaliana*, and *P. trichocarpa*) (Supplementary Table 1) were used to construct two unrooted phylogenetic trees using MEGA 7.0, respectively (Kumar et al., 2016). Neighbor-joining topologies were generated as the consensus of 1,000 bootstrap alignment replicates by running MEGA 7.0 with ClustalW alignment.

### Physical Localization and Gene Duplication Analysis of *PeINV*s

The chromosome localization data for each *INV* identified in moso bamboo were retrieved from the generic file format (GFF) file using TBtools 1.08. MCScanX software with the default settings was used to identify duplicated and syntenic *INV*s within the moso bamboo genome and those that were orthologous between moso bamboo and the *O. sativa*, *B. amplexicaulis*, or *O. latifolia* genomes (Wang et al., 2012). The chromosome distributions and syntenic relationships of the *INV*s were visualized using Circos 0.69-9 software (Krzywinski et al., 2009). The syntenic analysis maps were constructed using TBtools 1.08 software to display the syntenic relationships among the orthologous *INV*s in moso bamboo and other selected species (Chen et al., 2020), and the non-synonymous substitution (*Ka*), synonymous substitution (*Ks*), and *Ka*/*Ks* ratios of syntenic *INV* gene pairs were calculated using KaKs_Calculator 2.0 software (Wang et al., 2010).

### Promoter Sequence Analysis of *PeINV*s

The 2.0 kb genomic DNA sequences upstream of the translation start of *PeINV*s were retrieved from the moso bamboo database (Zhao et al., 2018). The PlantCARE^7^ database was used to search for *cis*-acting elements associated with abiotic stress and phytohormone-dependent responses within promoter regions (Lescot et al., 2002).

### RNA-Seq Data Analysis

The RNA-seq data for 26 tissues from moso bamboo (Zhao et al., 2018), which contained information about rhizomes, and the different growth stages of roots and shoots, leaves, and shoot buds (Sequence Read Archive accession number: SRS1847048–SRS1847073), were used for specific expression analysis of *PeINV*s. The RNA-seq data for moso bamboo leaves under drought stress (Huang et al., 2016a) were used to determine the expression patterns for *PeINV*s and *SWTG*s. The differentially expressed genes (DEGs) were identified using the Limma 3.13 software package in R 3.6.1 (Ritchie et al., 2015). The fragments per kilobase of transcript per million fragments mapped (FPKM) of these genes were extracted. The heatmap was drawn using the Pheatmap 1.0 package in R 3.6.1, with Euclidean distances and the complete linkage method for hierarchical clustering (Yang et al., 2017).

### Plant Materials and Sucrose, Glucose, and Fructose Contents Determination

Moso bamboo seedlings were grown in a climatic chamber under a 12 h light/12 h dark photoperiod at 25°C. The treatment consisted of subjecting 2-month-old seedlings to drought stress [simulated by 20% (w/v) polyethylene glycol-6000 (PEG-6000)]. At stipulated times, the leaves were immediately collected at 0, 1, 2, 4, or 8 h, frozen in liquid nitrogen and stored at −80°C. All samples were collected from at least three biological replicates.

The sucrose, glucose, and fructose contents in leaves were determined using a sucrose-glucose-fructose content (hexokinase method) determination kit (Suzhou Grace Biotechnology Co., Ltd., Jiangsu, China). Sucrose and fructose are converted to glucose by specific enzymes, and glucose is reduced to nicotinamide adenine dinucleotide phosphate (NADPH) to NADP^+^ by enzyme complexes. The sucrose, glucose, and fructose contents were calculated by measuring the increase in NADPH.

### RNA Isolation, qRT-PCR Analysis

Total RNA was extracted from the collected samples using an RNAprep Pure Plant kit (Tianmo, Beijing, China) according to the manufacturer’s instructions. First-strand cDNA was synthesized using a PrimeScript RT Reagent kit (Takara, Dalian, China). And qRT-PCR was performed using specific primers (Supplementary Table 2) designed by Primer Premier 5.0 software to check the DEGs identified by the RNA-seq data. *PeTIP41* was used as an internal control (Fan et al., 2013). The relative expression level of each gene was calculated using the 2^–ΔΔCT^ method with three technical repetitions (Livak and Schmittgen, 2001). The results were visualized using the Origin Pro 2017 software.

### Gene Cloning, Yeast Transformation, and Drought Tolerance Analysis

Specific primers were designed for the open reading frame (ORF) based on the nucleotide sequences for *PeCWINV8* in the bamboo genome database^8^ (Zhao et al., 2018; Supplementary Table 2). The cDNA was used as a template in the PCR process, which was performed using the primer pair PrimeSTAR Max mix (Takara, Japan). The PCR amplification products were inserted into the pGEM-T easy vector (Promega, Madison, WI, United States) and subsequently verified by sequencing (Ruibiotech, China). The PCR fragments were subcloned into the corresponding sites of the pYES2 vector (Invitrogen, Carlsbad, CA, United States), and then the vectors were introduced into INVSc1 yeast cells (Invitrogen, United States) using the transformation kit (Weidibio, China). Yeast cells expressing *PeCWINV8* along with control cells were grown on Yeast Extract Peptone Dextrose Medium (YPD) solid medium (1% yeast extract, 2% peptone, and 2% glucose). For the stress analysis, transformed yeast cells were propagated in SC-U medium containing 2% galactose for 12 h and the cell density was adjusted to 1.0 of OD_600_ followed by serial dilutions. Yeast cell were spotted on YPD medium supplemented with PEG-6000 (0, 1, and 2%). The plates were maintained at 30°C and growth was monitored after 2 days (Alavilli et al., 2016; Sun et al., 2021).

### Correlations and Co-expression Network Analyses

The correlations between the gene expression levels of 16 DEGs and the sugar contents of bamboo leaves under drought stress were constructed by calculating pairwise Pearson’s correlation coefficients (PCC) (Schober et al., 2018). Based on these correlations, a correlation heatmap of gene expression quantity and sugar content was generated using the Pheatmap 1.0 package in R 3.6.1. In addition, a co-expression network was constructed using Cytoscape 3.7.1 (Shannon et al., 2003) based on the results of the PCCs between *PeINV*s and *SWTG*s. The threshold for new edges was set with a PCC magnitude >0.8 and a *p*-value < 0.1, which is believed to indicate strongly co-expressed genes.

### Statistical Analysis

The statistical analyses were preformed using IBM SPSS Statistics 22.0 (Armonk, NY, United States), and the mean and standard deviation of three biological replicates were presented. Significant differences are indicated at ^∗^*p* < 0.05, ^∗∗^*p* < 0.01.

## Results

### Invertase Family Members in Moso Bamboo

To identify the INVs in moso bamboo, the rice and Arabidopsis INVs were used as queries subjected to a Basic Local Alignment Search Tool (BLASTN) search in moso bamboo database. A total of 29 PeINVs containing fully conserved INV domains were identified in moso bamboo. The INV family in plants can be classified into three major classes: NINVs, CWINVs, and VINVs, based on their sequence homology. Among the 29 PeINVs, 15 were neutral/alkaline INVs (PeNINVs), and 14 were acid INVs (PeAINVs), which consisted of 10 cell-wall INVs (PeCWINVs) and four vacuolar INVs (PeVINVs) (Table 1).

**TABLE 1:** Invertase family members in five different species.

	NINVs	CWINVs	VINVs	Total
*Phyllostachys edulis*	15	10	4	29
*Bonia amplexicaulis*	16	8	4	28
*Olyra latifolia*	8	7	2	17
*Oryza sativa*	8	9	2	19
*Arabidopsis thaliana*	9	6	2	17

**FIGURE 1:**
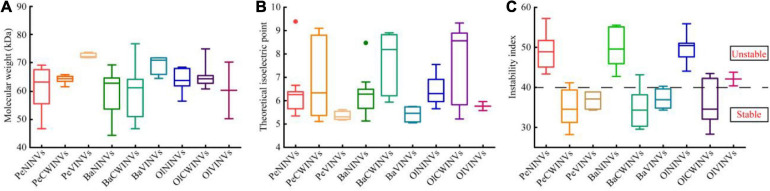
Amino acid characteristics of INVs in three bamboo species (Pe, Ba, and Ol indicate *Phyllostachys edulis*, *Bonia amplexicaulis*, and *Olyra latifolia*, respectively). **(A)** Molecular weight of proteins. **(B)** Theoretical isoelectric point (pI) of proteins. **(C)** Instability index of proteins.

We found that the protein molecular weight, theoretical isoelectric point, and protein instability index of the INV proteins varied dramatically among the three classes. However, they were relatively stable within the same class (Figure 1 and Supplementary Table 3). The protein molecular weight of the NINV class ranged from 46.7 to 69.4 kDa, those of the CWINV class ranged from 61.7 to 66.0 kDa, and those of the VINV class ranged from 71.7 to 73.7 kDa (Figure 1A and Supplementary Table 3). The theoretical isoelectric point (pI) of the NINV class ranged from 5.11 (PeNINV1) to 9.39 (PeNINV10), with an average of 6.32; those of the VINV class were all less than 6, but the CWINV class ranged from 5.11 (PeCWINV1) to 9.10 (PeCWINV5) with an average of 6.93 (Figure 1B and Supplementary Table 3). All the proteins in the NINV class were predicted to be unstable proteins with an instability index higher than 40. In contrast, 85% (12/14) of the CWINV and VINV classes were predicted to be stable proteins (Figure 1C and Supplementary Table 3).

Meanwhile, as the representative for different ploidy bamboo species, a hexaploid woody bamboo (*B. amplexicaulis*) and a diploid herbaceous bamboo (*O. latifolia*) were selected for comparative analysis. The result showed that 28 BaINVs and 17 OlINVs were discovered in *B. amplexicaulis* and *O. latifolia*, respectively, using the same procedure. They had similar classifications and characteristics as their orthologs in moso bamboo (Table 1 and Figure 1). The number of INV members in woody bamboo (*P. edulis* and *B. amplexicaulis*) was higher than that of the herbaceous plants (*O. latifolia*, *O. sativa*, and *A. thaliana*).

### Gene Structure Analysis of *PeINV*s

**FIGURE 2:**
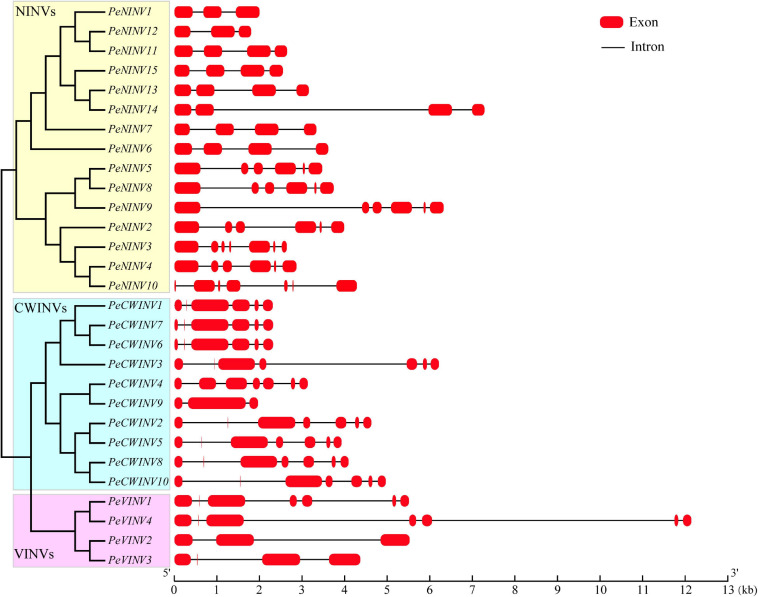
Gene structure of *PeINV*s.

Information about the distribution of exons and introns is vital when attempting to understand gene structure. Thus, we analyzed exon-intron features of 29 *PeINV*s, including the number and length of exons and introns (Figure 2 and Supplementary Table 4). The phylogenetic analysis revealed that the *AINV* subfamily members in moso bamboo could be divided into two clades, which were cell-wall-targeted (*CWINV*), and vacuole-targeted (*VINV*). The exon numbers for *PeCWINV*s ranged from 6 to 7, except for *PeCWINV9*, which had three exons. Four *PeVINV*s encoded irregular exons. Two contained seven exons, and the other two genes contained three or four exons. Interestingly, the first exon in *PeCWINV*s was shorter than that in *PeVINV*s, and 11 members of the *PeAINV*s encoded a mini-exon, which is one of the smallest exons in plants (Bournay et al., 1996). Many genes in the NINV subfamily are typically encoded by four or six exons in different classes (Nonis et al., 2008; Shen et al., 2018b; Bezerra-Neto et al., 2019). In line with previous reports, *PeNINV*s usually possess four or six exons, except *PeNINV1* and *PeNINV12* with three exons, and *PeNINV3* and *PeNINV10* with seven exons. In addition, seven members of the *PeNINV*s contained one shorter exon, as if it had been erroneously predicted by an intron or had originated within other exons. Most *PeINV*s clustered in the same group had similar numbers of exons and introns. Further analyses of the structural features of *INV*s in *B*. *amplexicaulis* and *O*. *latifolia* also supported this observation (Supplementary Figure 1).

### Distribution of Conserved Motifs in PeINVs

**FIGURE 3:**
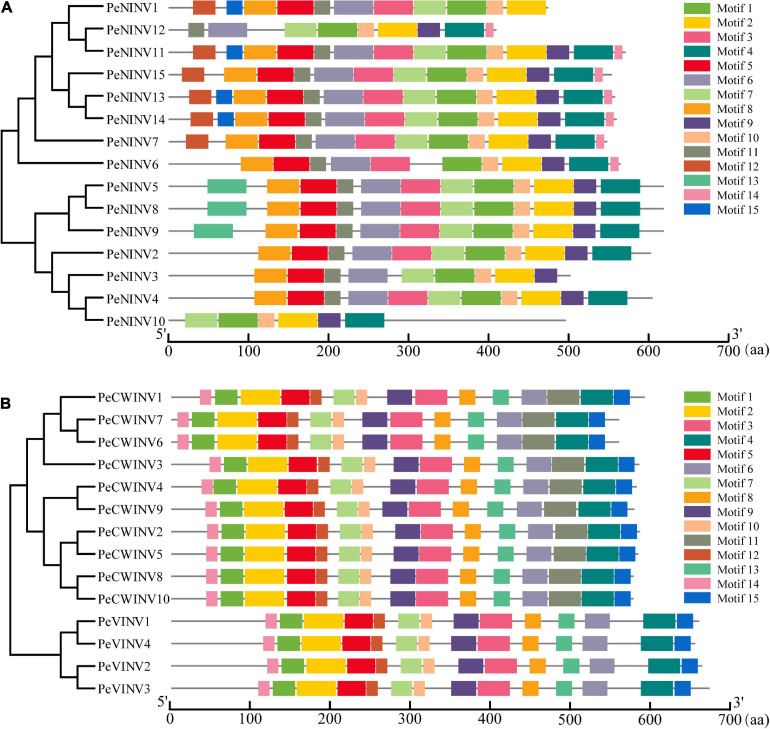
Distribution of motifs in PeINVs. **(A)** Motifs in PeNINVs. **(B)** Motifs in PeAINVs (PeCWINVs and PeVINVs). These motifs were investigated using the MEME web server and TBtools software. The different motifs were represented by different colors.

We employed the MEME web server combined with TBtools software to determine the motif type and location in different PeINVs. The results showed that 15 conserved motifs were identified in PeAINVs and PeNINVs (Figure 3). Among these AINV motifs, most motifs classified as CWINVs or VINVs were similar. However, compared to the CWINVs, the non-conserved sequences at the N-terminus of VINVs were longer, and motif 11 was specifically distributed in the CWINVs. The conserved motifs-NDPNG, WECP/YDF, and RDP were localized in motif 1, motif 10, and motif 12 (Figure 3A and Supplementary Figure 3B), respectively (Lammens et al., 2009).

The NINVs were conserved for the putative functional motifs of their orthologs in moso bamboo. In general, after excluding some NINVs lacking complete sequences (PeNINV1, PeNINV10, and PeNINV12), the paralogs of PeNINVs were generally conserved with regards to motif distribution, except for motif 12, motif 13, and motif 15 in the N-terminus as well as motif 14 in the C-terminus. Eleven motifs were observed to be consistent in their distributions and sizes, which included two motifs (motif 3 and motif 6) containing catalytic residues (Figure 3B and Supplementary Figure 3A; Itoh et al., 2004; Ji et al., 2005). Motif 14 was unique to eight PeINVs, motif 12 was specifically distributed in the N-terminus of six genes, motif 15 was specifically distributed in the N-terminus of four genes, and motif 13 was distributed in the N-terminus of three PeINVs. Further analyses of the motif features of INVs in *B. amplexicaulis* and *O. latifolia* produced results that were similar to the moso bamboo results (Supplementary Figure 2).

### Chromosome Localization, Gene Duplication, and Syntenic Analyses of *PeINV*s

**FIGURE 4:**
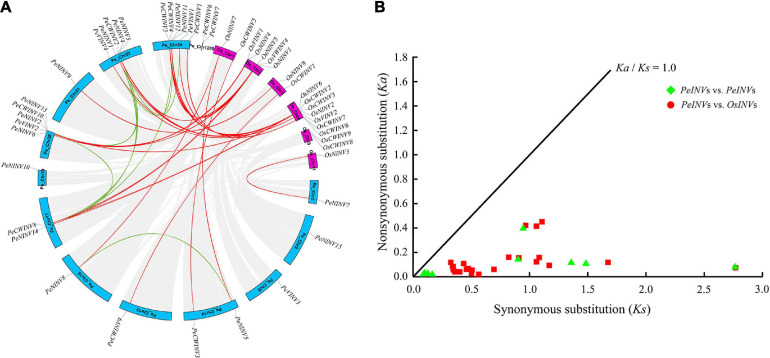
Synteny regions and *Ka*/*Ks* ratios analyses of INV genes. **(A)** Chromosome location and synteny regions of all *PeINV*s and *OsINV*s. The duplicated genes of *PeINV*s and *PeINV*s/*OsINV*s on different chromosomes were indicated with green and red lines, respectively. **(B)** The *Ka*/*Ks* ratios of INV homologous gene pairs.

The coordinates of 29 *PeINV*s were extracted from the moso bamboo GFF file to analyze their localization on the moso bamboo genome. All the *PeINV*s were distributed on 13 moso bamboo chromosomes at different densities (Figure 4A). Chromosome 24 contained the largest number of *PeINV*s (six), followed by chromosome 20 and chromosome 23 with five *PeINV*s, and one or two on the other chromosomes.

All moso bamboo and rice *INV*s were analyzed using the BLAST and MCScanX, and visualized using the basic functions in the TBtools software to investigate their syntenic status. In this study, nine segmental duplication pairs were found within *PeINV*s, and 25 segmental duplication pairs were detected in *PeINV*s/*OsINV*s, but no tandem duplication events were detected (Figure 4A). These results indicate that the driving force for the expansion of *INV*s in moso bamboo and rice was segmental duplication events.

To further investigate the evolutionary selection pressure on *INV*s in moso bamboo and rice, the *Ka*, synonymous substitution (*Ks*), and the *Ka*/*Ks* ratios of all segmental duplication pairs were calculated. The *Ka*/*Ks* values of 34 pairs of duplicated genes were all below 1.0 (Figure 4B and Supplementary Table 5), suggesting that the evolutionary selection pressure acting on *INV*s in moso bamboo tended to be purification. A further synteny analysis comparison of *PeINV*s/*OlINV*s, and *PeINV*s/*BaINV*s in moso bamboo with *B. amplexicaulis* and *O. latifolia* showed consistent results (Supplementary Figure 4 and Supplementary Table 5).

### Phylogenetic Analysis of INVs

**FIGURE 5:**
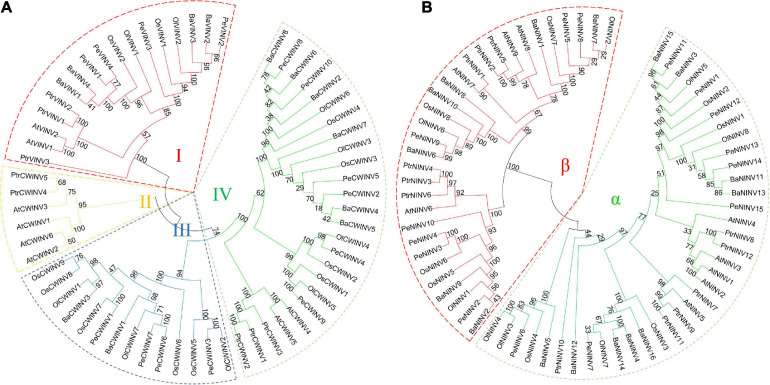
Phylogenetic tree of INVs in bamboo and other plants. **(A)** Acid invertase (CWINV and VINV). **(B)** Neutral/alkaline invertase (NINV). The phylogenetic tree was constructed via the neighbor-joining method (1,000 bootstrap replicates) using MEGA 7.0 software. The Pe, Ba, Ol, Os, At, and Ptr indicate invertase genes of *Phyllostachys edulis*, *Bonia amplexicaulis*, *Olyra latifolia*, *Oryza sativa*, *Arabidopsis thaliana*, and *Populus trichocarpa*, respectively.

To understand the evolutionary relationships among the INVs across different species, two unrooted phylogenetic trees were constructed for AINVs and NINVs using protein sequences from moso bamboo, *B. amplexicaulis*, *O. latifolia*, *O. sativa*, *A. thaliana*, and *P. trichocarpa* (Figure 5). Six phylogenetic trees were also constructed using INVs from these plant species, respectively (Supplementary Figure 5). A comparison of these phylogenetic trees demonstrated that the relationships among the INVs in six plant species were consistent and conserved. All INVs can be classified into AINV and NINV classes. AINVs can be divided into VINV and CWINV subgroups, and NINVs can be further subdivided into α and β subgroups (Figure 5).

The phylogenetic tree for the AINVs from six plant species formed four branches designated as branches I to IV (Figure 5A), and the members of different species in each branch were diverse. For example, branch I included four PeVINVs, branch III contained four PeCWINVs, and six PeCWINVs were clustered in branch IV. More than 70% (10/14) of PeAINVs from moso bamboo were clustered in branch I and branch IV, whereas only two and four members of rice were clustered in these two branches, respectively. All the members in branch I were VINVs and were found in six plant species, while those in branch II and branch III were only from dicots and monocots, respectively. The number of monocot members in branch IV was higher than the number of dicots.

The phylogenetic tree for the NINVs from six plant species could be separated into two distinct branches, referred to as the α and β branches (Figure 5B; Wang et al., 2017). There were eight and seven PeNINVs in the α and β branches, respectively. Moso bamboo also has more members (14) in the NINV subfamily than rice (eight), which may be related to the genomic replication events experienced during the evolution of moso bamboo. In the α branch, most NINVs from dicots were clustered together, suggesting that the gene duplication was more ancient in monocots than that in dicots (Wang et al., 2017).

### Expression Patterns of *PeINV*s in Different Tissues

**FIGURE 6:**
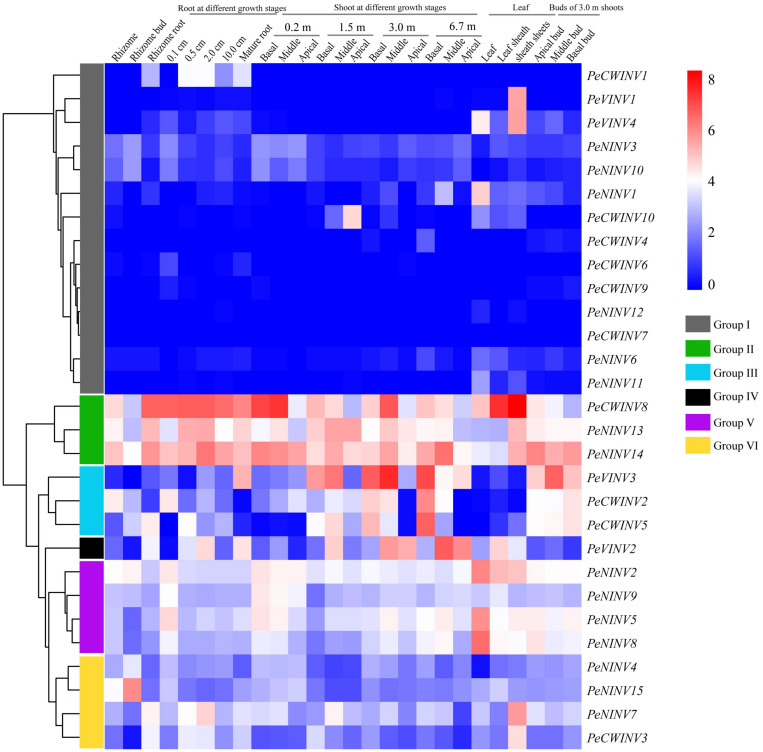
Expression patterns of 29 *PeINV*s in 26 different tissues based on RNA-seq data of moso bamboo. FPKM-normalized values from RNA sequence data of different tissues in the moso bamboo were used to construct the heatmap. The *PeINV*s were divided into six groups.

We used a published RNA-seq dataset of moso bamboo to investigate the expression patterns of *PeINV*s in diverse tissues. Twenty-nine *PeINV*s were divided into six groups based on the hierarchical clustering of expression patterns (Figure 6). There were 14 *PeINV*s in group I, most of which were scarcely expressed in 80% of the 26 tissues; group II contained three *PeINV*s expressed in every tissue, especially in the shoots and roots at different growth stages; and group III contained three *PeINV*s, which were preferentially expressed in shoots at different stages and in the 3.0 m shoot buds. These results also indicate that the *PeINV*s in group II and group III might be directly or indirectly involved in the rapid growth of bamboo shoots. Only *PeVINV2* in group IV, was mainly expressed in shoots and leaves. Group V included four *PeINV*s that were primarily detected in tender tissues (0.1 cm roots, 0.2 m shoots, leaves, and shoot buds). Four *PeINV*s in group VI were scarcely expressed in bamboo shoots and shoot buds, but were detected in the rhizomes and roots. These results suggest that *PeINV*s may play different roles in the growth of moso bamboo.

### Sugar Content and Expression of *PeINV*s and *SWTG*s in Bamboo Leaves Under Drought Stress

The glucose, fructose, and sucrose contents in bamboo leaves were investigated to understand the roles of *PeINV*s during drought stress (Supplementary Figure 6). The results showed that the glucose and fructose contents gradually increased with the prolonged treatment. The sucrose content also gradually increased, reached a maximum at 4 h, and then decreased at 8 h to a level that was similar to that at 2 h. Further analysis found that the fructose and glucose/sucrose ratio was about 70.0% at 0–4 h, but significantly increased to 93.7% at 8 h. These results indicated that the sugar content might be influenced by drought stress.

The promoter region analysis of *PeINV*s showed that a large number of *cis*-acting elements were associated with drought stress (Supplementary Figure 7), indicating that *PeINV*s might be involved in response to drought stress. INVs have previously been reported to act as significant regulators of water deficit (Ruan et al., 2010), and some studies have shown that *SWTG*s are induced under drought conditions (Zanor et al., 2009; Kelly et al., 2017). Therefore, the expression patterns of 182 genes in five gene families (29 *PeINV*s, 36 *PeSWEET*s, 19 *PePLT*s, 41 *PeSTP*s, and 57 *PeAQP*s) were analyzed using RNA-seq data generated from the leaves of moso bamboo under drought stress. As shown in Supplementary Figure 8, 16 upregulated DEGs (4 *PeINV*s and 12 *SWTG*s), were identified using FPKM (|log_2_ (fold change)| ≥ 1 and *p*-value < 0.05). The expression patterns of these genes in moso bamboo leaves under drought treatment (20% PEG-6000) were further validated by qRT-PCR.

**FIGURE 7:**
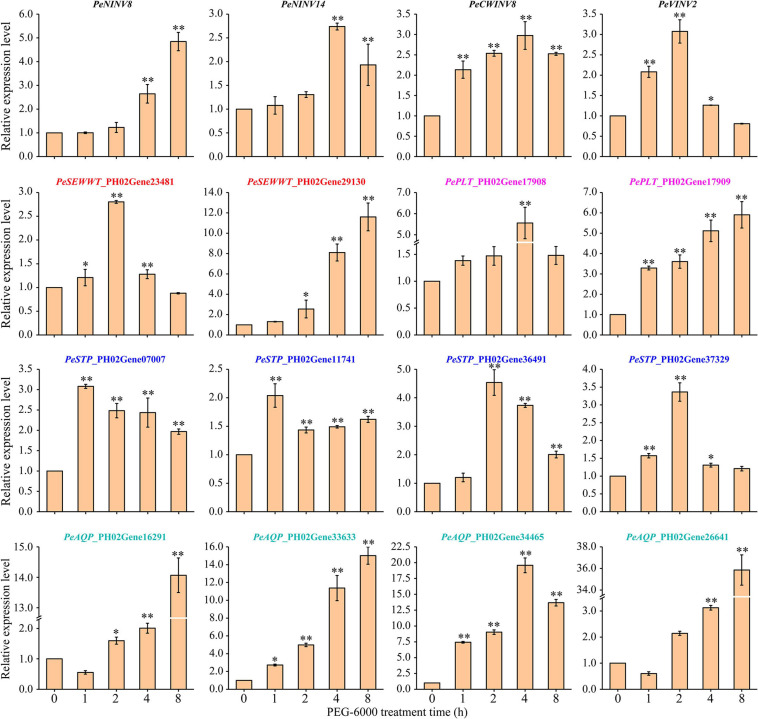
Expression patterns of 4 *PeINV*s and 12 *SWTG*s of moso bamboo leaves under drought stress. Fold-change values were used *PeTIP41* as internal controls with three technical replicates for each of three biological replicates in the qRT-PCR. The results are expressed as ±SD. The single and double asterisks indicate significant differences at 0.05 and 0.01 levels of the gene expression after different treated times according to student’s *t* test.

Overall, all 4 *PeINV*s and 12 *SWTG*s were upregulated with different patterns under drought stress (Figure 7). Four genes (*PeNINV8*, *PeSWEET*_PH02Gene29130, *PePLT*_PH02Gene179 09, and *PeAQP*_PH02Gene33633) continuously upregulated, which were significantly higher at the late stage of stress treatment (4 or 8 h) than those in the control (0 h). The expression of eight genes (*PeNINV14*, *PeCWINV8*, *PeVINV2*, *PeSWEET*_PH02Gene29130, *PePLT*_PH02Gene17908, *PeSTP*_P H02Gene36491, *PeSTP*_PH02Gene37329, and *PeAQP*_PH02G ene34465) increased at first, reached their highest expression level at 2 or 4 h and then decreased. In contrast, two water transport genes (*PeAQP*_PH02Gene16291 and *PeAQP*_PH02Gene26641) decreased at first and then increased, reaching their highest expression levels at 8 h. In addition, *PeSTP*_PH02Gene07007 and *PeSTP*_PH02Gene11741 expressions under drought stress were significantly higher than those in the control group (0 h), but their expression patterns fluctuated. These results suggest that the spatio-temporal expressions of *PeINV*s and *SWTG*s might play different roles in moso bamboo leaves under drought stress.

### Correlation Analyses of Sugar Contents With the Expression Levels of *PeINV*s and *SWTG*s

**FIGURE 8:**
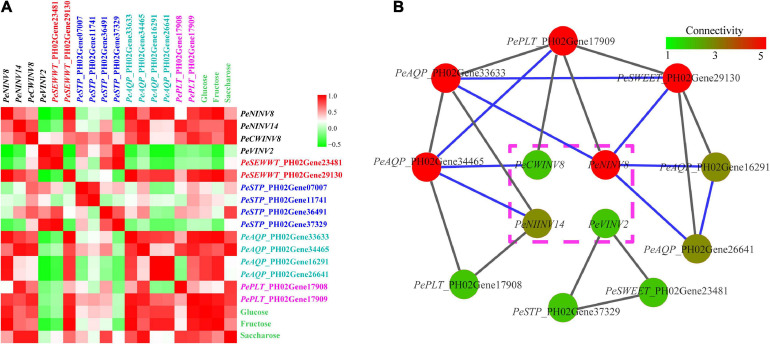
Correlation and co-expression analyses. **(A)** The correlation analysis of sugar contents with the expression levels of 4 *PeINV*s and 12 *SWTG*s. Each correlation is shown by the shades of green to red square shape. The gradual change color of green to red indicate negative to positive correlation. **(B)** Co-expression network of four *PeINV*s with nine *SWTG*s. Gray lines represent PCC magnitude from 0.8 to 0.9, and blue lines represent PCC magnitude from 0.9 to 1.0. The gradual change in the color of nodes represents the difference in the enriched number of co-expressed genes.

Based on the results of sugar content measurement and qRT-PCRs analysis, a PCC analysis was conducted. The results showed that the *PeNINV8* expression level had a significantly positive correlation with glucose and fructose contents, with PCC values of 0.911 and 0.982, respectively, and *PeINV14* and *PeCWINV*8 were positively correlated with sucrose content, with values of 0.906 and 0.917, respectively. Conversely, *PeVINV2* expression level was negatively correlated with glucose and fructose, with values of −0.208 and −0.441, respectively (Figure 8A and Supplementary Table 6). Furthermore, the expression levels of four *SWTG*s (*PeAQP*_PH02Gene33633, *PeAQP*_PH02Gene34465, *PePLT*_PH02Gene17909, and *PeSWEET*_PH02Gene29130) also showed positive correlations with sucrose, glucose or fructose contents. In addition, *PeINV*s were co-expressed with *SWTG*s, which suggested that they may be key genes related to sugar hydrolysis and transport in moso bamboo (Figure 8B). These results suggest that all of the above genes might respond to drought stress by influencing sugar content through gene expression.

### Validation of *PeINV*s Involved in Drought Stress

**FIGURE 9:**
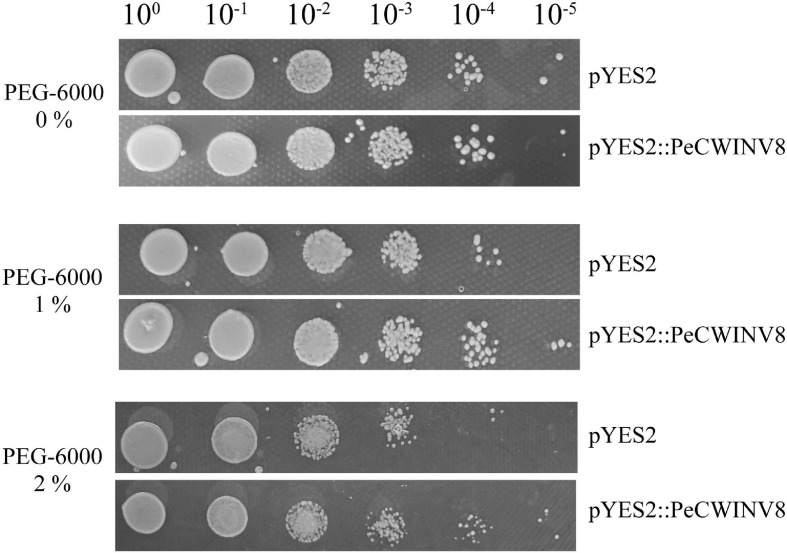
Effect of *PeCWNV8* expression on yeast under drought stress. Yeast cells with pYES2 vector only (pYES2) and yeast cells harboring *PeCWINV8* expressing construct (pYES2:PeCWINV8) were subjected to 1 or 2% PEG-6000. Cell density adjusted to OD_600_ at 1.0 and serial dilutions were made at each step. Five microliters of each dilution were inoculated on SC-U medium, SC-U medium supplemented with PEG-6000. Photographs were taken after 2 days of incubation at 30°C.

To validate the function of *PeINV*s involved in drought stress, *PeCWINV8* was selected and overexpressed in yeast cells, followed by PEG-6000 treatment. The result showed that both transgenic yeast cells harboring pYES2:PeCWINV8 and pYES2 vector could grow well on SC-U medium after 10^–5^ dilution, and two vectors grew slower on SC-U supplemented with 2% PEG-6000 than those on SC-U medium. The cells containing pYES2:PeCWINV8 were able to grow until 10^–5^ dilution, whereas those containing pYES2 vector only to 10^–4^ dilution on the SC-U medium supplemented with 1 or 2% PEG-6000 (Figure 9). These results indicated that overexpressed *PeCWINV8* can increase the drought tolerance of yeast cells, suggesting that *PeCWINV8* may be a key gene related to drought stress in moso bamboo.

## Discussion

Invertases are common in plants and play essential roles in vegetative development, seed germination, and fruit maturation (Sherson et al., 2003). Sugars and INVs play important roles in reproductive development under drought stress (Ruan et al., 2010). Therefore, a full understanding of the specific characteristics of INVs in different plant species will help us explore this particular function. A previous study reported that *PeAINV*s are involved in the internode elongation of moso bamboo shoots (Guo et al., 2020). However, no further information is available regarding the INV gene family in moso bamboo based on the updated genome (Zhao et al., 2018). To explore the INV gene family in moso bamboo, a comprehensive analysis of its gene structure, conserved motifs, evolutionary conservation, functional prediction, and expression patterns was conducted in this study. The results provide a basis for further function study of bamboo INVs.

### Conservation and Diversity of INVs

Many INVs have been identified in various plants over the past decades, and both monocotyledonous and dicotyledonous diploid plants contain no less than 13 members (Supplementary Table 1). In the present study, 29 members were identified in moso bamboo, which was more than the numbers identified in the diploid plants investigated in this study (19 in *A. thaliana*, 17 in *O. sativa*, and 17 in *O. latifolia*). Gene duplication and divergence play essential roles in the expansion of gene families and novel gene function during evolution (Jaillon et al., 2007). The moso bamboo genome had undergone at least one round of whole-genome duplication, followed by multiple segment duplication. These events are thought to be the reason for the expansion of INV family members during evolution (Peng et al., 2013). Gene duplication and syntenic analyses of *INV*s supported this assumption. A total of nine segmental duplication pairs were found within the *PeINV*s, and 25 segmental duplication pairs were detected between *PeINV*s and *OsINV*s (Figure 4A). Nevertheless, all 34 duplicated gene pairs had *Ka*/*Ks* < 1 (Figure 4B and Supplementary Table 5), indicating that *PeINV*s had undergone negative selection pressures with limited functional divergence after duplication (Hurst, 2002).

In the AINV sub-family, the PeAINVs contained NDPNG, WECP/VDF, and RDP motifs (Figure 3A and Supplementary Figure 3B). The NDPNG motif is partly encoded by a mini-exons, one of the smallest exons known to date in plants (Bournay et al., 1996), and it contributes the tripeptide DPN to the first conserved motif of AINVs in moso bamboo (Figure 3B). The phylogenetic analysis revealed that all AINVs could be separated into VINV and CWINV subgroups (Figure 5A and Supplementary Figure 5). The WECP/VDF motif contains two subgroups with different amino acids (a proline in the VINVs and a valine in the CWINVs), which might be responsible for the different enzyme activities (Alberto et al., 2004). The NINVs in bamboo could be classified into α and β branches based on 68 NINVs from six plant species (Figure 5B), which was consistent with the evolutionary characteristics of INVs (Roitsch and González, 2004). The PeNINVs in the α branch had similar protein lengths and had the same number of exons (Figure 2). However, the PeNINVs in the β branch were distinct from those in the α branch and formed two subgroups (Figure 5B; Pan et al., 2019). The diversity of *PeINV*s suggests that they might have undergone different evolutionary selections in moso bamboo.

### Multiple Functions of *INV*s Involved in Plants Growth and Development

The *INV*s are essential for plant growth and development (Ruan, 2014). Recent studies have shown that *CWINV*s play a critical role in flowering (Zanor et al., 2009), and seed and fruit development (Li et al., 2012; Wang and Ruan, 2012). Furthermore, *VINV*s are essential for hexose accumulation (Yamada et al., 2007) and cell expansion (Wang et al., 2014). High INV activity could result in the generation of a sugar concentration gradient in the phloem to facilitate sucrose unloading from source organs to sink organs (Qian et al., 2016). In maize (*Z. mays*), CWINVs have been implicated in early floral development, especially ovary expansion and seed filling (Cheng et al., 1996; Chourey et al., 2012). The detailed expression patterns for *INV*s have been studied in many other plant species (Ji et al., 2005; Bezerra-Neto et al., 2019), but the possible function of *PeINV*s remains unclear. Gene expression patterns are an important manifestation of gene functions. Multiple expression patterns for *PeINV*s were observed in different tissues of moso bamboo based on the RNA-seq data (Figure 6), indicating that they had different functions.

*PeNINV13* and *PeNINV14* were expressed in all tissues and were highly expressed in shoots and roots during the growth stage. This result was consistent with the homologous NINV gene in rice, which has been reported to play a role in carbon and energy supply during early root development (Yao et al., 2009). The expression levels of *PeCWINV2*, *PeCWINV5*, and *PeVINV2* were higher in immature shoots and buds than in other tissues, indicating that they have a potential role in tissue growth and development (Dahro et al., 2016). *PeCWINV1* and *PeVINV1* were significantly expressed in the roots or sheath sheets, and might be involved in cell wall modification (Ru et al., 2017). In contrast, the expression of some *PeINV*s was not detected at any stage, suggesting that they might be pseudogenes, are functionally redundant, or might be expressed in other specific tissues or under particular conditions (Wang and Ruan, 2012; Tauzin et al., 2014). *PeCWINV4*, *PeCWINV6*, *PeCWINV7*, *PeCWINV9*, and *PeNINV12* expressions were almost undetectable in 26 tissues. These results indicate that the 29 *PeINV*s might function differently in moso bamboo. In addition, the *PeNINV* genes were more abundant than the *PeAINV* genes, suggesting that *PeNINV*s might play more significant roles in moso bamboo growth.

### *INV*s Combined With Multiple Genes Involved in Response to Drought Stress

It has been reported that a double subcellular location gene (*PtrA*/*NINV*) can be upregulated by dehydration, cold, and salt stress (Dahro et al., 2016). A/N-Invs might help maintain the balance of reactive oxygen species (ROS) in mitochondria. Alternatively, they may or reduce oxidative damage and maintain the balance between adenosine diphosphate (ADP) and adenosine triphosphate (ATP) production under oxidative stress, most likely by delivering glucose as a substrate to hexokinase (Xiang et al., 2011). A previous study showed that sugar transporter pathway genes might play important roles in the response to stress (Wang et al., 2013). Sucrose is cleaved into two hexose molecules by VINVs, which facilitates water flow by doubling the osmotic action (Ruan et al., 2010). Hexokinase can influence sugar levels by regulating water conductance controlled by the expression of *AQP*s (Kelly et al., 2017; Zhang et al., 2019). Furthermore, *INV*s are also affected by other genes (such as *BFN1*, *PLD1*, and *CCP1*) in response to drought stress (McLaughlin and Boyer, 2004; Ruan et al., 2010). The RNA-seq and qRT-PCR results showed that the expression levels of the four *PeINV*s were significantly affected by drought stress (Supplementary Figure 8 and Figure 7). The upregulated expression of *PeINV*s in response to drought stress was probably due to more INVs being required to cleave sucrose into hexose sugars, which subsequently provided cells with more energy to sustain increased respiration activity (Sturm, 1999). The sugar content changes in moso bamboo leaves and overexpressed *PeCWINV8* in yeast cells under drought stress all supported this assumption (Supplementary Figure 6 and Figure 9).

Twelve *SWTG*s were detected as positive factors in response to drought stress (Figure 7). Nevertheless, gene expression is a dynamic process, and it is possible that most genes are transcriptionally activated only at a particular time point until the enzymes exert their functions (Dubrovina et al., 2013). Thus, the different expression patterns of the 12 *SWTG*s indicated that they might perform their function spatiotemporally under drought stress. Together, both the gene expression patterns and the sugar content changes could further improve our understanding about the co-expression network between *PeINV*s and sugar signaling pathways under drought stress (Wang et al., 2017). The co-expression network showed that four *PeINV*s co-expressed with nine *SWTG*s (Figure 8 and Supplementary Table 6), in which *PeNINV8* and *PeNINV14* may contribute the most to the NINV family when responding to drought stress. These results indicate that PeINVs, PeAQPs, and sugar transporters might work together in response to drought stress by modulating cellular osmotic potential (Ruan et al., 2010). However, the precise regulatory mechanisms of the *PeINV* and *SWTG* cascade in moso bamboo under drought stress require further investigation.

## Conclusion

This study has identified 29 *PeINV*s in moso bamboo. A sequence comparison of the paralogous gene analyses revealed that *PeINV*s had been subjected to stronger functional constraints during evolution. The PeINVs were both relatively conservative and diverse, with different expression patterns in different tissues and at different moso bamboo growth stages. Based on the sugar content correlation analyses and the expression levels of *PeINV*s and *SWTG*s in moso bamboo leaves, we propose that *PeINV*s might positively work together with *SWTG*s in response to drought stress. Drought regulation in plants is a complex network, and more work is required to define the function of *PeINV*s and elucidate their molecular mechanisms in moso bamboo under drought stress.

## Data Availability Statement

The datasets presented in this study can be found in online repositories. The names of the repository/repositories and accession number(s) can be found in the article/Supplementary Material.

## Author Contributions

ZG conceived and designed the experiments. CZ performed the experiments. KY analyzed the data. GL and YL contributed reagents, materials, and analysis tools. CZ, KY, and ZG wrote the manuscript. All authors contributed to the article and approved the submitted version.

## Conflict of Interest

The authors declare that the research was conducted in the absence of any commercial or financial relationships that could be construed as a potential conflict of interest.

## Publisher’s Note

All claims expressed in this article are solely those of the authors and do not necessarily represent those of their affiliated organizations, or those of the publisher, the editors and the reviewers. Any product that may be evaluated in this article, or claim that may be made by its manufacturer, is not guaranteed or endorsed by the publisher.

## Footnotes

1
http://bamboo.bamboogdb.org/#/

2
http://pfam.xfam.org/

3
http://smart.embl-heidelberg.de/

4
http://gsds.cbi.pku.edu.cn/

5
https://web.expasy.org/protparam/

6
http://meme-suite.org/tools/meme

7
http://bioinformatics.psb.ugent.be/webtools/plantcare/html/

8
http://www.bamboogdb.org

## Abbreviations

ADP: adenosine diphosphate
AINV: acid invertase
AQP: aquaporin
ATP: adenosine triphosphate
*AtINV*s: *Arabidopsis thaliana* invertase genes
*BaINV*s: *Bonia amplexicaulis* invertase genes
BFN1: bifunctional nuclease 1
bHLH: basic Helix–Loop–Helix transcription factor
BLASTP: basic local alignment tool for protein
CCP1: cysteine protease 1
cDNA: complementary deoxyribonucleic acid
CWINV: cell-wall invertase
DEG: differentially expressed gene
FPKM: fragments per kilobase of transcript per million fragments mapped
FW: fresh weight
GFF: generic file format
HMM: hidden Markov model
INV: invertase
*Ka*: non-synonymous substitution
kDa: kilodalton
*Ks*: synonymous substitution
LEA: late embryogenesis abundant
NADPH: nicotinamide adenine dinucleotide phosphate
NINV: alkaline/neutral invertase
*OsINV*s: *Oryza sativa* invertase genes
*OlINV*s: *Olyra latifolia* invertase genes
PCC: Pearson’s correlation coefficient
*PeAINV*s: *Phyllostachys edulis* acid invertase genes
*PeCWINV*s: *Phyllostachys edulis* cell-wall invertase genes
PEG-6000: polyethylene glycol-6000
*PeNINV*s: *Phyllostachys edulis* alkaline/neutral invertase genes
*PeINV*s: *Phyllostachys edulis* invertase genes
*PeVINV*s: *Phyllostachys edulis* vacuole invertase genes
pI: theoretical isoelectric point
PLD1: phospholipase D
PLT: polyol/monosaccharide transporter
*PtrINV*s: *Populus trichocarpa* invertase genes
qRT-PCR: quantitative real-time PCR
ROS: reactive oxygen species
SC-U: SC dropout medium without uracil
SMART: simple modular architecture research tool
STP: sugar transporter protein
SuSy: sucrose synthase
SWEET: sugars will eventually be exported transporter
SWTGs: sugar and water transport genes
UGE: UDP-galactose-4-epimerase
VINV: vacuole invertase
YPD: Yeast Extract Peptone Dextrose Medium
ZEP: zeaxanthin epoxidase.
