# Supplementary material for: Identification and Expression Analyses of Invertase Genes in Moso Bamboo Reveal Their Potential Drought Stress Functions
Source: Front Genet. 2021 Aug 30;12:696300. doi: 10.3389/fgene.2021.696300 (PMC8435750; doi:10.3389/fgene.2021.696300)
Supplement: Supplementary file 1 [file Data_Sheet_1.zip › Supplementary Material/Supplementary Figure.docx]

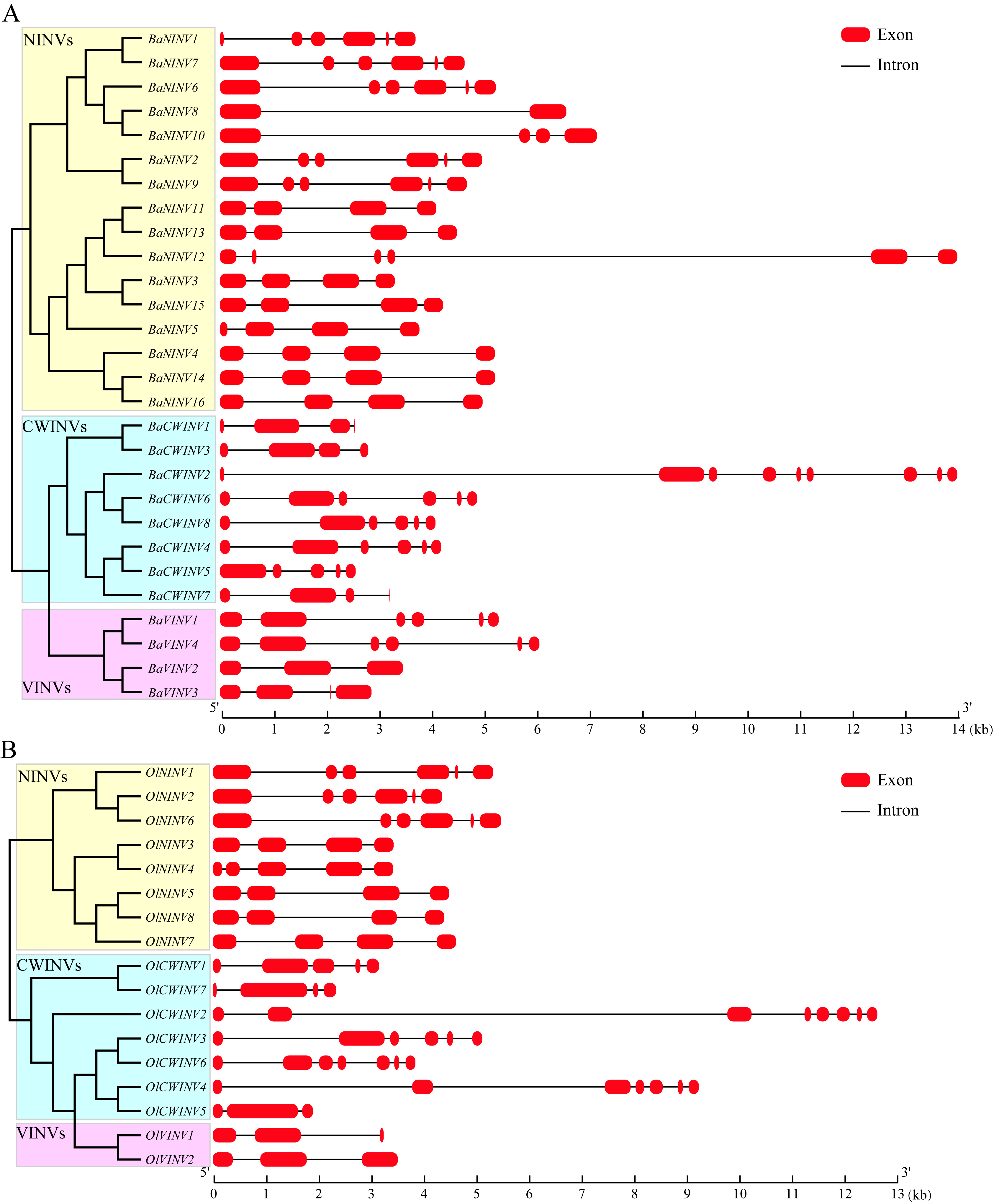


**Supplementary Figure 1. Gene structure of *BaINV*s in *Bonia amplexicaulis* (A), and *OlINV*s in *Olyra latifolia* (B).**


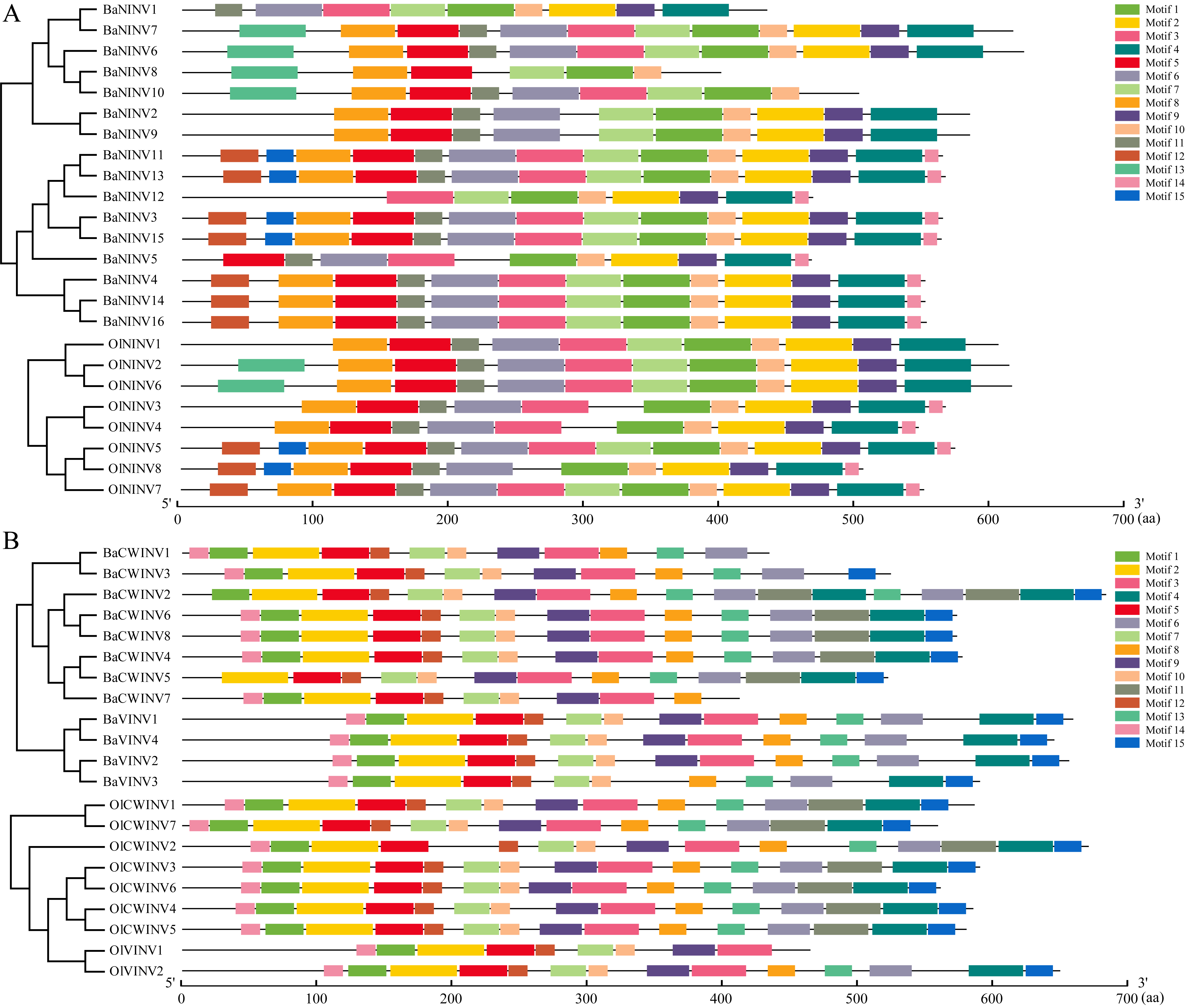


**Supplementary Figure 2. Motif distribution of INVs in *Bonia amplexicaulis* and *Olyra latifolia*.** (A) Motifs of BaNINVs and OlNINVs, (B) Motifs of BaAINVs (BaCWINVs and BaVINVs) and OlAINVs (OlCWINVs and OlVINVs).


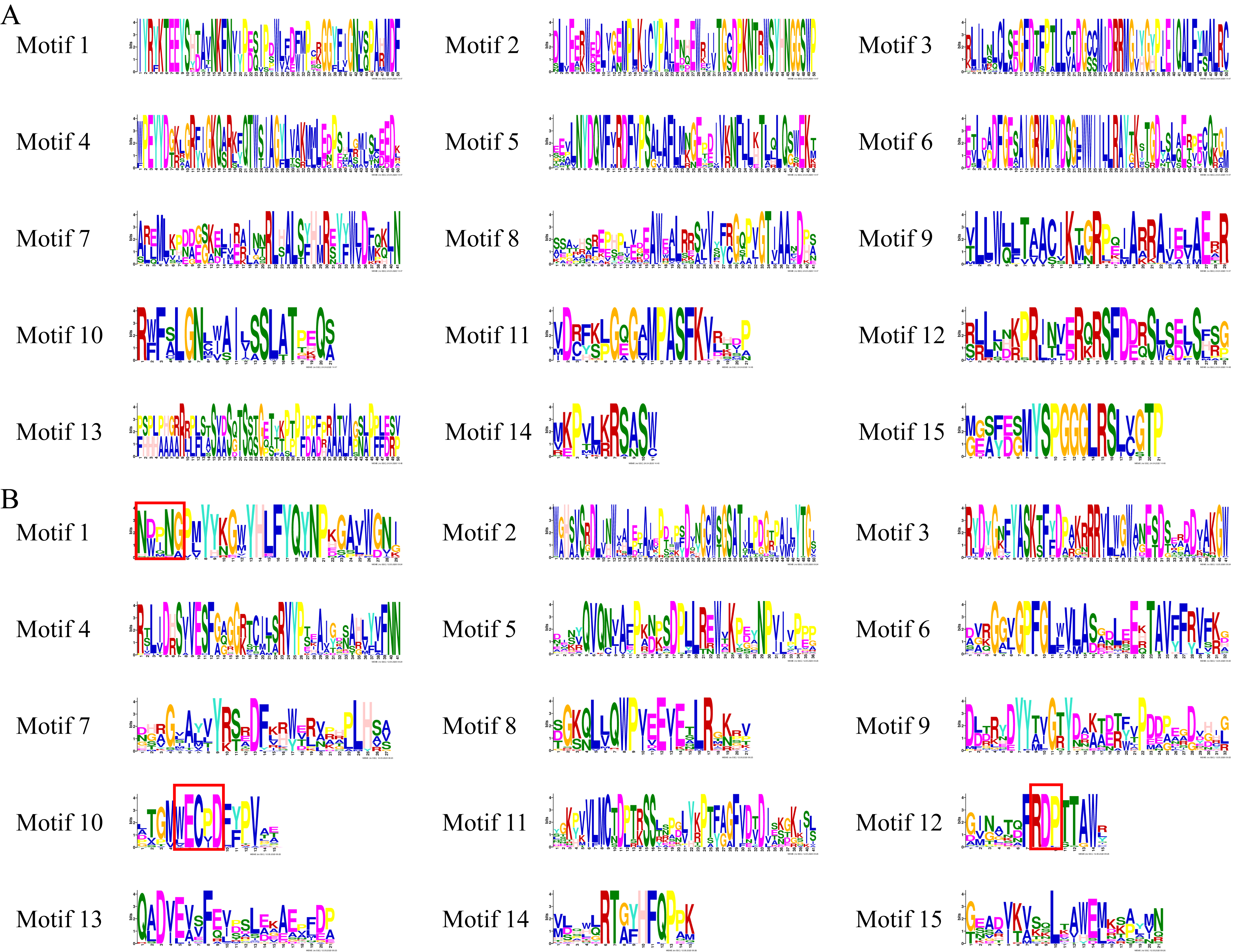


**Supplementary Figure 3. Motif logo sequences in NINVs and AINVs.** (A) Motif logo sequences of NINVs, (B) Motif logo sequences of AINVs.


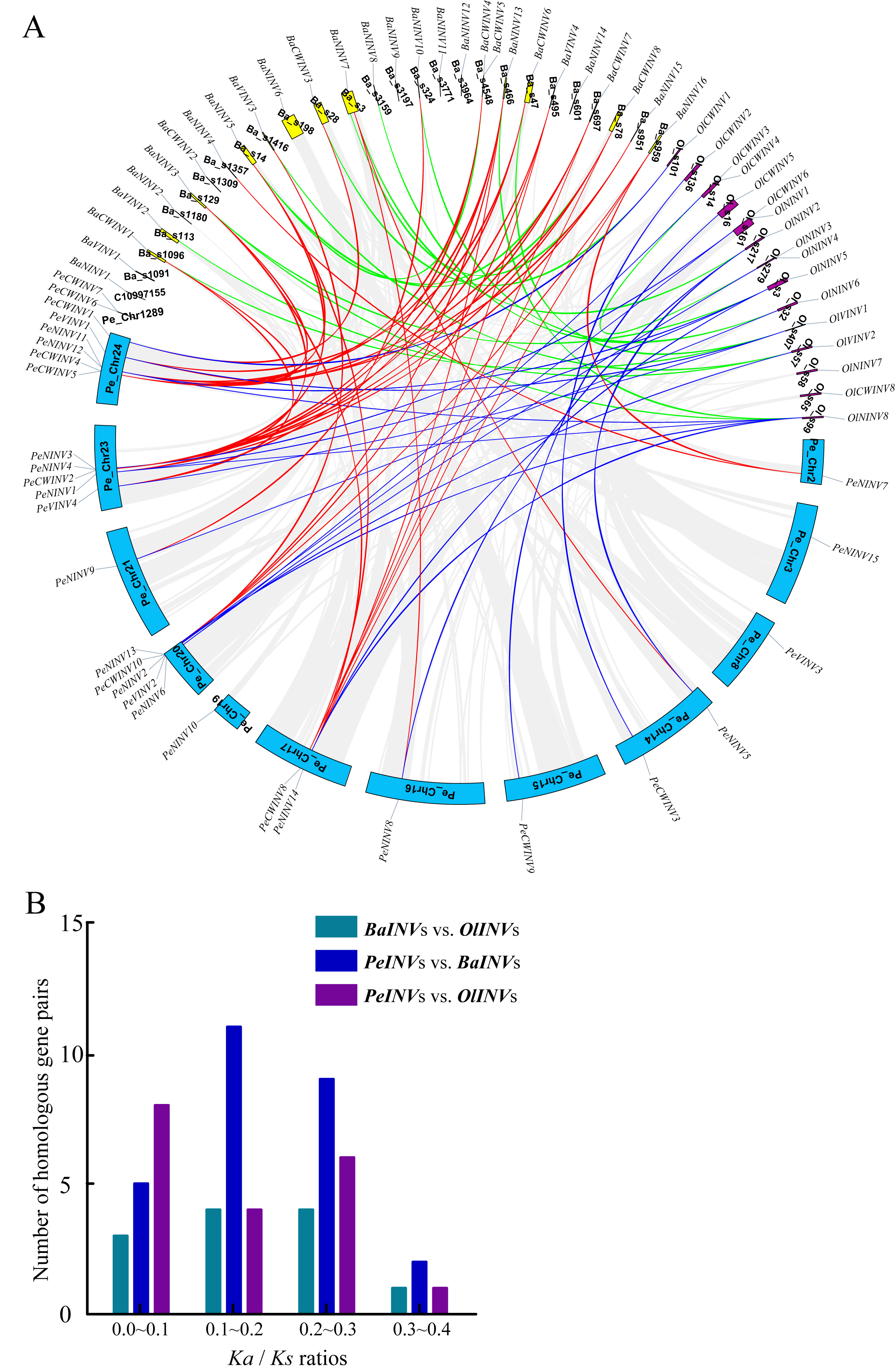


**Supplementary Figure 4. Synteny regions and *Ka*/*Ks* ratios of homologous gene pair of *INV*s.** (A) Synteny regions of *BaINV*s/*OlINV*s, *PeINV*s/*BaINV*s and *PeINV*s/*OlINV*s. The duplications genes of *BaINV*s/*OlINV*s, *PeINV*s/*BaINV*s and *PeINV*s/*OlINV*s on different scaffolds were indicated with green, red and blue lines, respectively. (B) The *Ka* / *Ks* ratios of INVs homologous gene pairs. Cyan, blue, and violet bar chart represented the homologous gene pairs of *BaINV*s/*OlINV*s, *PeINV*s/*BaINV*s and *PeINV*s/*OlINV*s.


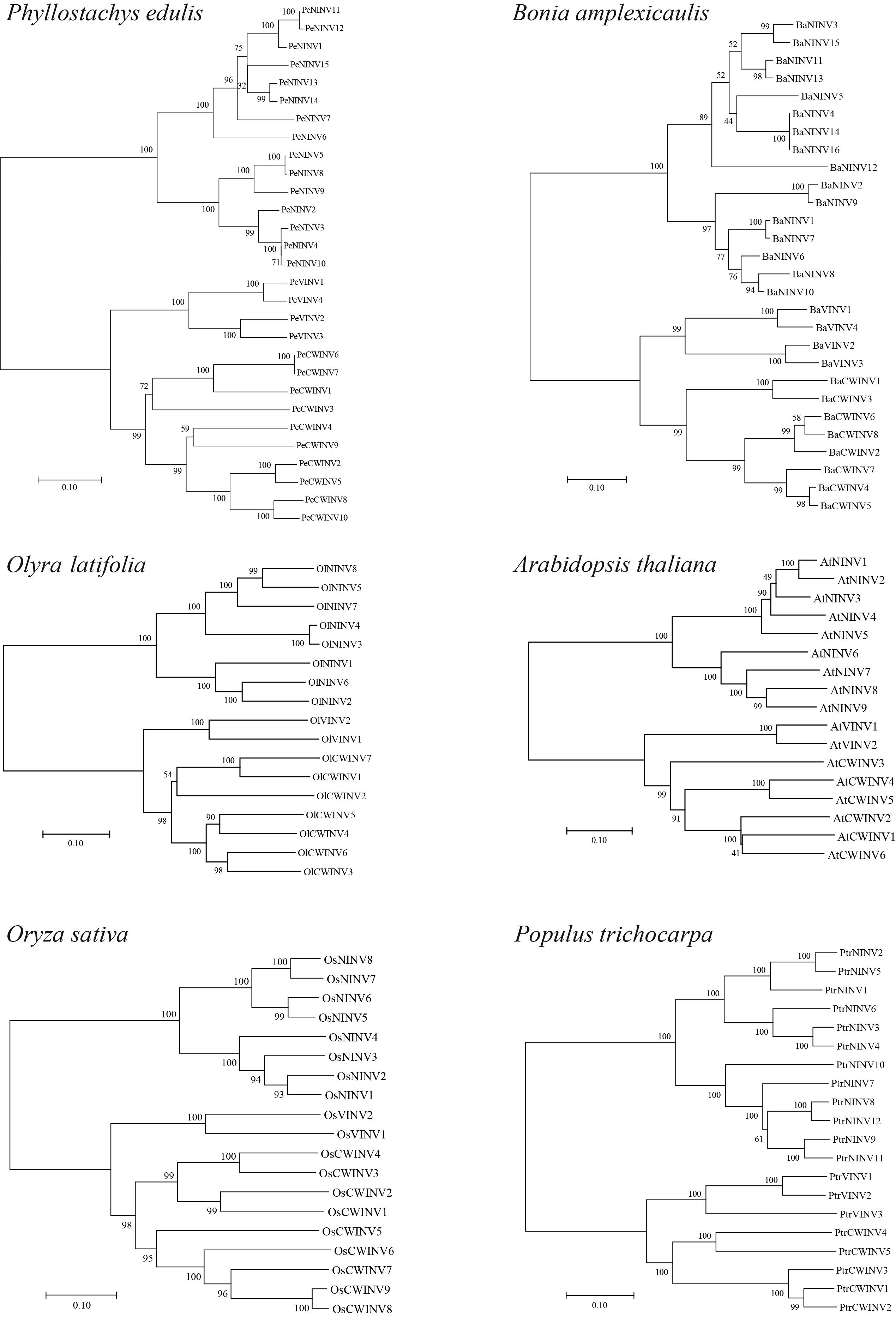


**Supplementary Figure 5. Phylogenetic tree of INVs in representative monocots (*Phyllostachys edulis, Bonia amplexicaulis, Olyra latifolia* and *Oryza sativa)* and dicots (*Arabidopsis thaliana* and *Populus trichocarpa*).**


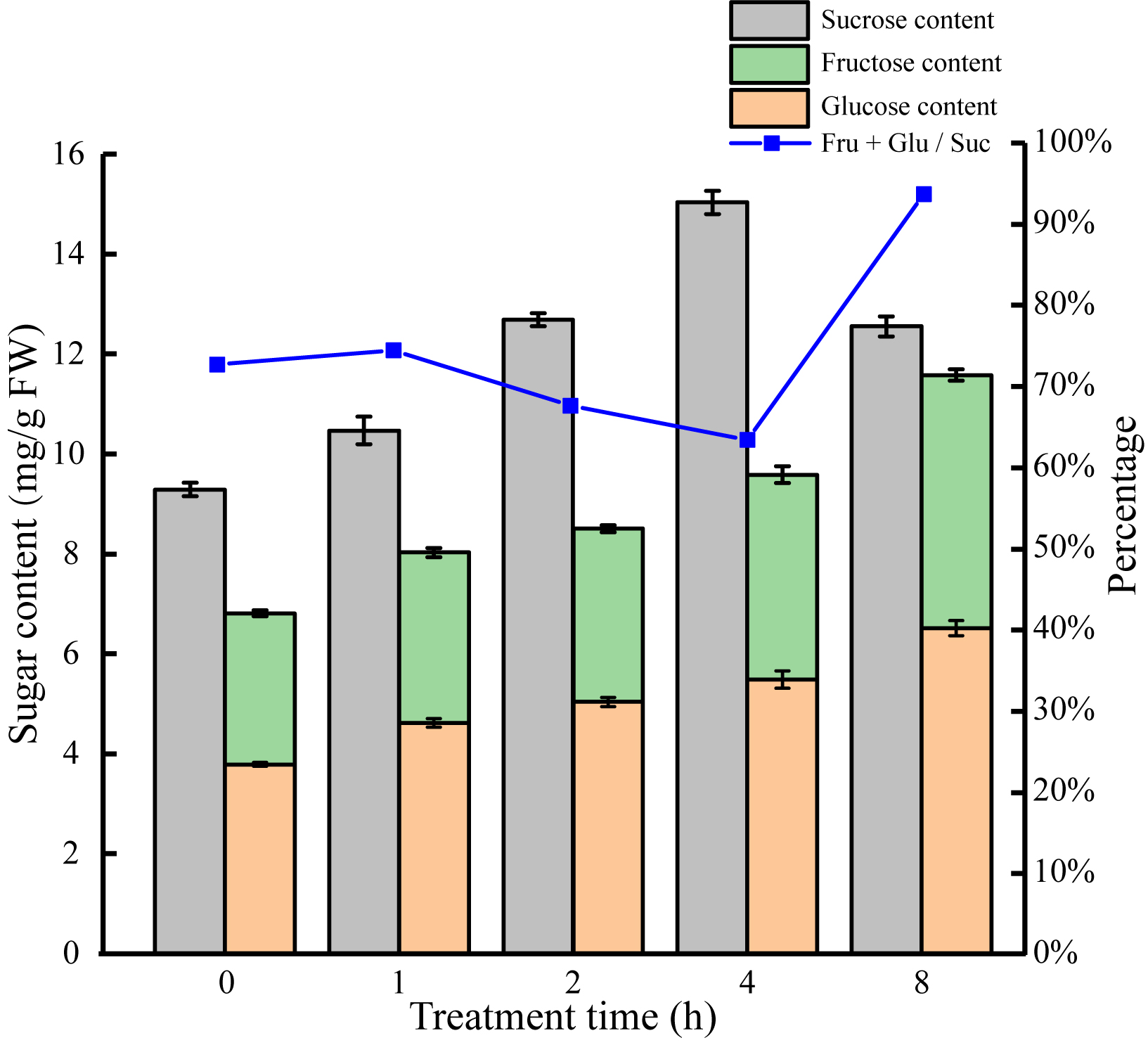


**Supplementary Figure 6. Sugar contents of bamboo leaves under drought stress treatment time at 0, 1, 2, 4, and 8 h.** The changes of sucrose, fructose, and glucose contents. FW: Fresh Weight.


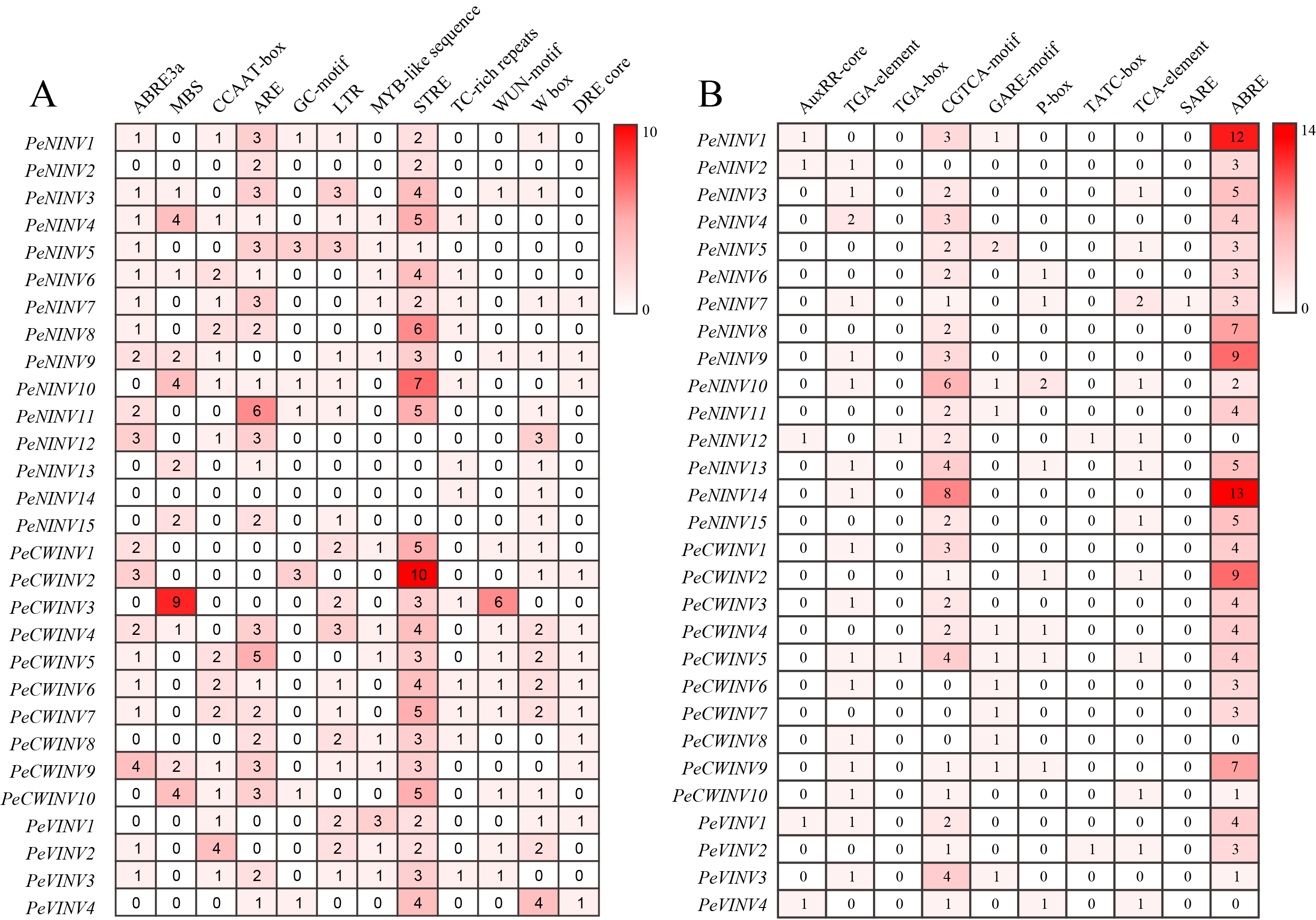


**Supplementary Figure 7. Distribution characteristics of stress-related and phytohormone-responsive *cis*-acting elements in *PeINV*s promoters.** Stress-related elements (A) and phytohormone-responsive elements (B) in the 2.0 kb promoters of *PeINV*s were collected by PlantCARE database.


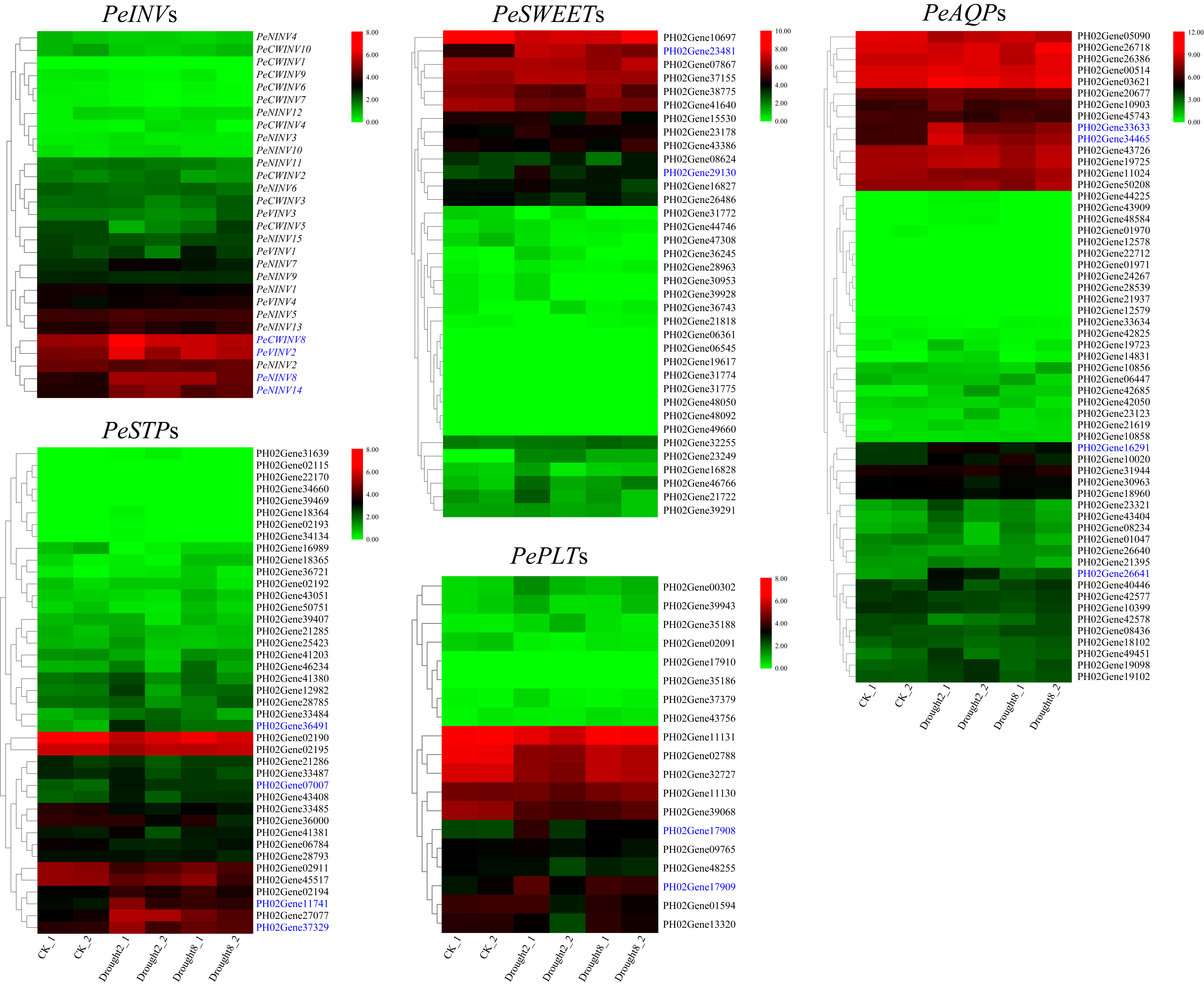


**Supplementary Figure 8. Expression heatmap of *PeINV*s, *PeSWEET*s, *PePLT*s, *PeSTP*s, *PeHXK*s and *PeAQP*s in moso bamboo leaves under drought stress by RNA-Seq data.** The blue font serial number of genes represented DEGs which were identified by Limma package in the R software.
