# Supplementary material for: Identification and Expression Analyses of Invertase Genes in Moso Bamboo Reveal Their Potential Drought Stress Functions
Source: Front Genet. 2021 Aug 30;12:696300. doi: 10.3389/fgene.2021.696300 (PMC8435750; doi:10.3389/fgene.2021.696300)
Supplement: Supplementary file 1 [file Data_Sheet_1.zip › Supplementary Material/Supplementary Table 2.docx]

**Supplementary Table 2. Primer sequences of qRT-PCR and gene cloning**

| Gene name | Forward sequence (5′—3′) | Reverse sequence (5′—3′) |
| --- | --- | --- |
| *PeNINV8* | GTGCTCTTCCCGGACACTTTC | CTCAGCAACCTCCACGCTTC |
| *PeNINV14* | TGATGAACGGCGAACCGG | ACCAGAACCCCGAATCTACCG |
| *PeCWINV8* | TCTCGACACGTCCAAGTTCG | CGTATCGCTCCGTCTTGTTGTT |
| *PeVINV2* | GAAGAGTAACAACGCCGAGGAG | GGTCGTAGAATGTCTTGGAGGC |
| *PeSWEET*_PH02Gene23481 | CTCTACATCACCATCCCCAACG | GTTCTTGCCATCGACGACGA |
| *PeSWEET*_PH02Gene29130 | GCTCAACTTCGAGAAATTAAGCC | GCAAACGACAATGACAGTAGGAT |
| *PePLT*_PH02Gene17908 | GACATGAGCAAGCTGTTCGG | CTTCAACTCCTATGGCACGGT |
| *PePLT*_PH02Gene17909 | TGCCTCCTTGCCACATAGAA | TCTGATCTGCTCCGTGCTTG |
| *PeSTP*_PH02Gene07007 | GAGATGGTGCTCGTCTGGAA | TTGGCGGACCCGAACTT |
| *PeSTP*_PH02Gene11741 | CCTGTTCCTGTCGGAGATCG | GGTGCCGTAGTTGACGAGGT |
| *PeSTP*_PH02Gene36491 | CTCATCCCCAGCGAGACCTT | AATCTGTAGTCTCATTGAGGTGGC |
| *PeSTP*_PH02Gene37329 | CCCTCATCTCCATCTTCACCG | GCGTACCCTTTGGGCATCTC |
| *PeAQP*_PH02Gene16291 | GCGGAAGCTGTCCCTCGT | TGCTGGAAACCCTTGACGAC |
| *PeAQP*_PH02Gene33633 | TGTTCATGGTCCACTTGGCG | GATGATAGGGCCGACCCAGA |
| *PeAQP*_PH02Gene34465 | TCGATCCATCCATTACTCCTCCT | CATCGCCAACAAACAGATGACA |
| *PeAQP*_PH02Gene26641 | TGACGACAGATCAGCGACACTG | AAGAGGAAGGTGAGCACGAGCT |
| *PeTIP41*  *ORF-PeCWINV8* | AAAATCATTGTAGGCCATTGTCG  ATGGGGACTCTGCAATGGGTC | ACTAAATTAAGCCAGCGGGAGTG  TTAGGCGCCATTCATCAGAGG |

**Supplementary Table 3. Putative basic physical and chemical characteristics of PeINVs, BaINVs and OlINVs.**

| Amino acid name | Gene assembly name | Protein size (aa) | Molecular weight (kDa) | Theoretical isoelectric point | Instability index | Ortholog in rice | Ortholog in Arabidopsis |
| --- | --- | --- | --- | --- | --- | --- | --- |
| PeNINV1 | PH02Gene05233.t1 | 475 | 53.04362 | 5.35 | 45.62 | OsNINV2 | AtNINV5 |
| PeNINV2 | PH02Gene05714.t2 | 604 | 67.80156 | 6.35 | 45.07 | OsNINV5 | AtNINV6 |
| PeNINV3 | PH02Gene06169.t1 | 503 | 55.65831 | 5.90 | 54.11 | OsNINV6 | AtNINV6 |
| PeNINV4 | PH02Gene06174.t1 | 606 | 67.35523 | 6.66 | 50.47 | OsNINV6 | AtNINV6 |
| PeNINV5 | PH02Gene08042.t1 | 620 | 69.39640 | 5.53 | 51.72 | OsNINV7 | AtNINV9 |
| PeNINV6 | PH02Gene10235.t1 | 566 | 64.35201 | 6.38 | 48.89 | OsNINV4 | AtNINV4 |
| PeNINV7 | PH02Gene10514.t1 | 549 | 62.84926 | 6.26 | 57.21 | OsNINV3 | AtNINV3 |
| PeNINV8 | PH02Gene19574.t1 | 620 | 69.40745 | 5.66 | 50.48 | OsNINV7 | AtNINV9 |
| PeNINV9 | PH02Gene19699.t1 | 620 | 69.44722 | 5.66 | 52.10 | OsNINV8 | AtNINV9 |
| PeNINV10 | PH02Gene21855.t1 | 497 | 55.52679 | 9.39 | 44.44 | OsNINV6 | AtNINV6 |
| PeNINV11 | PH02Gene27967.t1 | 572 | 63.90046 | 6.22 | 45.00 | OsNINV2 | AtNINV5 |
| PeNINV12 | PH02Gene27972.t1 | 410 | 46.77447 | 6.63 | 43.37 | OsNINV2 | AtNINV5 |
| PeNINV13 | PH02Gene30661.t1 | 559 | 63.01851 | 6.30 | 48.10 | OsNINV1 | AtNINV5 |
| PeNINV14 | PH02Gene31027.t1 | 561 | 63.19153 | 6.13 | 46.89 | OsNINV1 | AtNINV5 |
| PeNINV15 | PH02Gene34390.t1 | 555 | 63.39773 | 6.31 | 50.88 | OsNINV1 | AtNINV5 |
| PeCWINV1 | PH02Gene00392.t1 | 590 | 64.37626 | 5.11 | 40.19 | OsCWINV7 | AtCWINV6 |
| PeCWINV2 | PH02Gene06193.t1 | 584 | 65.40538 | 9.07 | 39.09 | OsCWINV3 | AtCWINV4 |
| PeCWINV3 | PH02Gene06876.t1 | 583 | 66.00063 | 6.09 | 41.15 | OsCWINV5 | AtCWINV6 |
| PeCWINV4 | PH02Gene16851.t1 | 580 | 65.55743 | 8.05 | 29.73 | OsCWINV2 | AtCWINV5 |
| PeCWINV5 | PH02Gene16852.t1 | 582 | 64.94969 | 9.10 | 38.04 | OsCWINV3 | AtCWINV5 |
| PeCWINV6 | PH02Gene20563.t1 | 558 | 61.71981 | 5.37 | 34.56 | OsCWINV9 | AtCWINV6 |
| PeCWINV7 | PH02Gene20565.t1 | 558 | 61.71981 | 5.37 | 34.56 | OsCWINV9 | AtCWINV6 |
| PeCWINV8 | PH02Gene26446.t1 | 576 | 64.30424 | 8.72 | 28.22 | OsCWINV3 | AtCWINV5 |
| PeCWINV9 | PH02Gene27247.t1 | 577 | 64.43565 | 5.79 | 31.80 | OsCWINV2 | AtCWINV5 |
| PeCWINV10 | PH02Gene30701.t1 | 576 | 64.78357 | 6.58 | 34.31 | OsCWINV3 | AtCWINV5 |
| PeVINV1 | PH02Gene02057.t1 | 658 | 72.12241 | 5.32 | 34.34 | OsVINV2 | AtVINV1 |
| PeVINV2 | PH02Gene05689.t1 | 662 | 72.19208 | 5.62 | 35.36 | OsVINV1 | AtVINV2 |
| PeVINV3 | PH02Gene09580.t1 | 671 | 73.69271 | 5.30 | 38.86 | OsVINV1 | AtVINV2 |
| PeVINV4 | PH02Gene09732.t1 | 653 | 71.71486 | 5.17 | 38.88 | OsVINV2 | AtVINV1 |
| BaNINV1 | Bam001043.1 | 432 | 49.36965 | 5.63 | 47.60 | OsNINV7 | AtNINV8 |
| BaNINV2 | Bam005147.1 | 582 | 65.28975 | 6.58 | 43.40 | OsNINV5 | AtNINV6 |
| BaNINV3 | Bam006808.1 | 562 | 63.19061 | 6.30 | 42.74 | OsNINV2. | AtNINV4 |
| BaNINV4 | Bam007340.1 | 549 | 62.67894 | 6.10 | 55.26 | OsNINV3 | AtNINV5 |
| BaNINV5 | Bam007666.1 | 465 | 53.17541 | 6.80 | 49.86 | OsNINV4 | AtNINV5 |
| BaNINV6 | Bam015155.1 | 622 | 69.53336 | 5.55 | 54.93 | OsNINV8 | AtNINV7 |
| BaNINV7 | Bam024009.1 | 614 | 68.66634 | 5.58 | 52.41 | OsNINV7 | AtNINV8 |
| BaNINV8 | Bam025610.1 | 398 | 44.37133 | 5.80 | 51.09 | OsNINV8 | AtNINV7 |
| BaNINV9 | Bam025666.1 | 582 | 65.36886 | 6.36 | 45.38 | OsNINV5 | AtNINV6 |
| BaNINV10 | Bam026459.1 | 500 | 55.62522 | 5.13 | 49.32 | OsNINV8 | AtNINV7 |
| BaNINV11 | Bam029287.1 | 562 | 63.34788 | 6.30 | 49.00 | OsNINV1 | AtNINV4 |
| BaNINV12 | Bam030011.1 | 466 | 53.16450 | 8.48 | 55.18 | OsNINV3 | AtNINV5 |
| BaNINV13 | Bam034196.1 | 564 | 63.37995 | 6.42 | 48.19 | OsNINV2 | AtNINV4 |
| BaNINV14 | Bam038656.1 | 549 | 62.69297 | 6.10 | 55.56 | OsNINV3 | AtNINV5 |
| BaNINV15 | Bam046364.1 | 561 | 63.05138 | 6.52 | 45.34 | OsNINV2 | AtNINV4 |
| BaNINV16 | Bam046618.1 | 550 | 62.94855 | 6.26 | 55.37 | OsNINV3 | AtNINV5 |
| BaCWINV1 | Bam003797.1 | 434 | 48.72056 | 5.94 | 36.83 | OsCWINV7 | AtCWINV1 |
| BaCWINV2 | Bam007033.1 | 693 | 77.06056 | 6.91 | 30.71 | OsCWINV4 | AtCWINV5 |
| BaCWINV3 | Bam021914.3 | 524 | 58.07534 | 5.98 | 38.61 | OsCWINV8 | AtCWINV1 |
| BaCWINV4 | Bam032618.1 | 577 | 64.40611 | 8.88 | 34.36 | OsCWINV3 | AtCWINV5 |
| BaCWINV5 | Bam032619.1 | 522 | 58.65138 | 8.68 | 34.32 | OsCWINV3 | AtCWINV5 |
| BaCWINV6 | Bam035627.1 | 573 | 64.08896 | 8.32 | 29.56 | OsCWINV4 | AtCWINV5 |
| BaCWINV7 | Bam039826.1 | 412 | 46.75805 | 8.91 | 43.16 | OsCWINV3 | AtCWINV5 |
| BaCWINV8 | Bam041578.1 | 573 | 64.26507 | 8.07 | 30.15 | OsCWINV4 | AtCWINV5 |
| BaVINV1 | Bam003971.1 | 659 | 72.07111 | 5.05 | 40.31 | OsVINV2 | AtVINV1 |
| BaVINV2 | Bam004818.1 | 656 | 71.93669 | 5.71 | 36.30 | OsVINV1 | AtVINV2 |
| BaVINV3 | Bam008116.1 | 590 | 64.66971 | 5.75 | 34.35 | OsVINV1 | AtVINV2 |
| BaVINV4 | Bam034484.1 | 645 | 70.44639 | 5.21 | 37.59 | OsVINV2 | AtVINV1 |
| OlNINV1 | Ola007832.1 | 604 | 67.84767 | 6.90 | 47.12 | OsNINV5 | AtNINV6 |
| OlNINV2 | Ola013911.1 | 612 | 68.38007 | 5.66 | 50.77 | OsNINV7 | AtNINV8 |
| OlNINV3 | Ola018025.1 | 565 | 63.89864 | 6.92 | 50.13 | OsNINV4 | AtNINV5 |
| OlNINV4 | Ola018029.1 | 545 | 61.77625 | 7.55 | 51.09 | OsNINV4 | AtNINV5 |
| OlNINV5 | Ola019342.1 | 572 | 63.97473 | 6.41 | 44.06 | OsNINV2 | AtNINV4 |
| OlNINV6 | Ola020740.1 | 614 | 68.67928 | 6.12 | 50.85 | OsNINV8 | AtNINV7 |
| OlNINV7 | Ola031357.1 | 549 | 62.74512 | 5.91 | 55.91 | OsNINV3 | AtNINV5 |
| OlNINV8 | Ola039050.2 | 504 | 56.58694 | 6.21 | 49.08 | OsNINV1 | AtNINV4 |
| OlCWINV1 | Ola000897.1 | 583 | 64.54179 | 5.96 | 43.49 | OsCWINV8 | AtCWINV1 |
| OlCWINV2 | Ola004900.1 | 667 | 75.22971 | 8.89 | 42.24 | OsCWINV5 | AtCWINV1 |
| OlCWINV3 | Ola005595.1 | 587 | 65.38957 | 9.33 | 37.41 | OsCWINV3 | AtCWINV5 |
| OlCWINV4 | Ola005596.1 | 582 | 65.69960 | 8.57 | 28.30 | OsCWINV2 | AtCWINV5 |
| OlCWINV5 | Ola007632.1 | 577 | 64.23247 | 5.83 | 32.08 | OsCWINV1 | AtCWINV5 |
| OlCWINV6 | Ola007783.1 | 558 | 62.91776 | 8.88 | 34.57 | OsCWINV4 | AtCWINV5 |
| OlCWINV7 | Ola033474.1 | 556 | 60.93506 | 5.22 | 32.41 | OsCWINV7 | AtCWINV1 |
| OlVINV1 | Ola025141.1 | 462 | 50.32879 | 5.58 | 43.81 | OsVINV2 | AtVINV2 |
| OlVINV2 | Ola031057.1 | 646 | 70.52945 | 5.96 | 40.39 | OsVINV1 | AtVINV1 |

**Supplementary Table 5. The nonsynonymous substitution (*Ka*), synonymous substitution (*Ks*), and *Ka*/*Ks* ratios of homologous gene pair of *INV*s.**

| Gene_1 | Gene_2 | Nonsynonymous substitution (*Ka*) | Synonymous substitution (*Ks*) | Nonsynonymous/synonymous (*Ka*/*Ks*) |
| --- | --- | --- | --- | --- |
| *PeCWINV8* | *PeCWINV2* | 0.395712 | 0.946567 | 0.418049 |
| *PeNINV1* | *PeNINV11* | 0.025838 | 0.128785 | 0.200631 |
| *PeNINV1* | *PeNINV13* | 0.114301 | 1.359221 | 0.084093 |
| *PeNINV14* | *PeNINV1* | 0.107054 | 1.484898 | 0.072095 |
| *PeNINV14* | *PeNINV12* | 0.077594 | 2.764980 | 0.028063 |
| *PeNINV14* | *PeNINV13* | 0.018971 | 0.088377 | 0.214659 |
| *PeNINV4* | *PeNINV2* | 0.141574 | 0.902864 | 0.156806 |
| *PeNINV5* | *PeNINV8* | 0.018282 | 0.165373 | 0.110548 |
| *PeVINV4* | *PeVINV1* | 0.029904 | 0.102180 | 0.292656 |
| *PeCWINV1* | *OsCWINV7* | 0.108234 | 0.434897 | 0.248872 |
| *PeCWINV2* | *OsCWINV4* | 0.160807 | 0.821395 | 0.195774 |
| *PeCWINV3* | *OsCWINV5* | 0.053158 | 0.506503 | 0.104952 |
| *PeCWINV5* | *OsCWINV4* | 0.155310 | 0.907127 | 0.171210 |
| *PeCWINV8* | *OsCWINV4* | 0.414972 | 1.059448 | 0.391687 |
| *PeCWINV8* | *OsCWINV2* | 0.421126 | 0.967488 | 0.435278 |
| *PeCWINV9* | *OsCWINV1* | 0.450318 | 1.107054 | 0.406772 |
| *PeNINV1* | *OsNINV2* | 0.061643 | 0.458509 | 0.134442 |
| *PeNINV11* | *OsNINV1* | 0.092543 | 1.169083 | 0.079159 |
| *PeNINV12* | *OsNINV1* | 0.073023 | 2.768481 | 0.026377 |
| *PeNINV12* | *OsNINV2* | 0.060089 | 0.694746 | 0.086491 |
| *PeNINV14* | *OsNINV1* | 0.025902 | 0.501896 | 0.051608 |
| *PeNINV14* | *OsNINV2* | 0.118173 | 1.673964 | 0.070594 |
| *PeNINV2* | *OsNINV5* | 0.040305 | 0.401257 | 0.100448 |
| *PeNINV2* | *OsNINV6* | 0.122374 | 1.059152 | 0.115539 |
| *PeNINV4* | *OsNINV5* | 0.157639 | 1.083104 | 0.145544 |
| *PeNINV4* | *OsNINV6* | 0.084665 | 0.338463 | 0.250145 |
| *PeNINV5* | *OsNINV7* | 0.041775 | 0.371943 | 0.112316 |
| *PeNINV6* | *OsNINV4* | 0.067010 | 0.475307 | 0.140982 |
| *PeNINV7* | *OsNINV3* | 0.019281 | 0.564554 | 0.034152 |
| *PeNINV8* | *OsNINV7* | 0.040205 | 0.359850 | 0.111726 |
| *PeNINV9* | *OsNINV8* | 0.055192 | 0.345884 | 0.159569 |
| *PeVINV1* | *OsVINV2* | 0.083222 | 0.344183 | 0.241797 |
| *PeVINV2* | *OsVINV1* | 0.117871 | 0.325324 | 0.362319 |
| *PeVINV4* | *OsVINV2* | 0.081877 | 0.344736 | 0.237506 |
| *PeVINV1* | *BaCWINV1* | 0.036407 | 0.132642 | 0.274475 |
| *PeVINV4* | *BaCWINV1* | 0.035624 | 0.139605 | 0.255180 |
| *PeCWINV1* | *BaCWINV3* | 0.137143 | 0.421950 | 0.325022 |
| *PeCWINV2* | *BaCWINV4* | 0.039649 | 0.239107 | 0.165822 |
| *PeCWINV2* | *BaCWINV6* | 0.145820 | 0.726915 | 0.200601 |
| *PeCWINV8* | *BaCWINV6* | 0.037441 | 0.225534 | 0.166012 |
| *PeCWINV2* | *BaCWINV7* | 0.029138 | 0.157761 | 0.184697 |
| *PeCWINV8* | *BaCWINV7* | 0.181759 | 0.642128 | 0.283056 |
| *PeCWINV8* | *BaCWINV8* | 0.032582 | 0.197567 | 0.164915 |
| *PeNINV9* | *BaNINV10* | 0.020666 | 0.107433 | 0.192364 |
| *PeNINV1* | *BaNINV13* | 0.107479 | 1.340250 | 0.080193 |
| *PeNINV12* | *BaNINV13* | 0.079128 | 2.215658 | 0.035713 |
| *PeNINV13* | *BaNINV13* | 0.019579 | 0.063826 | 0.306758 |
| *PeNINV14* | *BaNINV13* | 0.013150 | 0.105733 | 0.124367 |
| *PeNINV1* | *BaNINV15* | 0.021031 | 0.135308 | 0.155427 |
| *PeNINV12* | *BaNINV15* | 0.016660 | 0.154982 | 0.107496 |
| *PeNINV13* | *BaNINV16* | 0.157545 | - | - |
| *PeNINV14* | *BaNINV16* | 0.154636 | - | - |
| *PeNINV7* | *BaNINV16* | 0.013950 | 0.191964 | 0.072671 |
| *PeNINV1* | *BaNINV3* | 0.028145 | 0.116676 | 0.241219 |
| *PeNINV12* | *BaNINV3* | 0.017233 | 0.160406 | 0.107433 |
| *PeNINV14* | *BaNINV3* | 0.086847 | 1.348435 | 0.064406 |
| *PeNINV7* | *BaNINV4* | 0.011946 | 0.169985 | 0.070278 |
| *PeNINV6* | *BaNINV5* | 0.016899 | 0.083095 | 0.203374 |
| *PeNINV5* | *BaNINV7* | 0.017979 | 0.149399 | 0.120345 |
| *PeNINV8* | *BaNINV7* | 0.017387 | 0.145687 | 0.119344 |
| *PeVINV2* | *BaVINV3* | 0.046890 | 0.171309 | 0.273717 |
| *PeVINV1* | *BaVINV4* | 0.036615 | 0.131300 | 0.278862 |
| *PeVINV4* | *BaVINV4* | 0.041350 | 0.143531 | 0.288091 |
| *PeCWINV1* | *OlCWINV1* | 0.168451 | 0.471692 | 0.357120 |
| *PeCWINV2* | *OlCWINV3* | 0.089698 | 0.366902 | 0.244473 |
| *PeCWINV8* | *OlCWINV3* | 0.193408 | 0.699361 | 0.276550 |
| *PeCWINV9* | *OlCWINV5* | 0.072096 | 0.250265 | 0.288078 |
| *PeNINV2* | *OlCWINV5* | 0.023034 | 0.182269 | 0.126372 |
| *PeNINV4* | *OlCWINV5* | 0.130621 | 1.047232 | 0.124730 |
| *PeNINV5* | *OlNINV2* | 0.017444 | 0.227058 | 0.076825 |
| *PeNINV8* | *OlNINV2* | 0.017098 | 0.23058 | 0.074152 |
| *PeNINV6* | *OlNINV3* | 0.057794 | 0.258058 | 0.223956 |
| *PeNINV6* | *OlNINV4* | 0.052664 | 0.217146 | 0.242528 |
| *PeNINV1* | *OlNINV5* | 0.033589 | 0.227288 | 0.147780 |
| *PeNINV12* | *OlNINV5* | 0.029917 | 0.337768 | 0.088572 |
| *PeNINV14* | *OlNINV5* | 0.099220 | 1.627832 | 0.060952 |
| *PeNINV9* | *OlNINV6* | 0.031466 | 0.181426 | 0.173437 |
| *PeNINV1* | *OlNINV8* | 0.114738 | 1.233199 | 0.093041 |
| *PeNINV12* | *OlNINV8* | 0.077010 | 2.504902 | 0.030744 |
| *PeNINV13* | *OlNINV8* | 0.020274 | 0.224114 | 0.090465 |
| *PeNINV14* | *OlNINV8* | 0.009597 | 0.237642 | 0.040383 |
| *PeVINV2* | *OlVINV2* | 0.079463 | 0.286222 | 0.277626 |
| *BaVINV2* | *OlVINV2* | 0.083677 | 0.326077 | 0.256618 |
| *BaNINV3* | *OlNINV5* | 0.033691 | 0.261102 | 0.129034 |
| *BaNINV3* | *OlNINV8* | 0.088006 | 1.238906 | 0.071035 |
| *BaNINV5* | *OlNINV4* | 0.042026 | 0.246956 | 0.170175 |
| *BaVINV3* | *OlVINV2* | 0.086040 | 0.328926 | 0.261579 |
| *BaNINV8* | *OlNINV6* | 0.074815 | 0.272343 | 0.274709 |
| *BaNINV13* | *OlNINV8* | 0.010474 | 0.218791 | 0.047874 |
| *BaCWINV3* | *OlCWINV1* | 0.060941 | 0.189834 | 0.321021 |
| *BaCWINV7* | *OlCWINV3* | 0.060519 | 0.337110 | 0.179523 |
| *BaCWINV8* | *OlCWINV3* | 0.184673 | 0.784244 | 0.235479 |
| *BaNINV7* | *OlNINV2* | 0.013420 | 0.218904 | 0.061305 |
| *BaNINV15* | *OlNINV5* | 0.027561 | 0.249114 | 0.110635 |

**Supplementary Table 6. The Pearson’s correlation coefficients (PCC) of** **sugar contents and the gene expression levels of *PeINVs* and *SWTGs* under drought stress.**

| Group_A | Group_B | Correlation coefficients | *P*-values |
| --- | --- | --- | --- |

| *PeAQP*_PH02Gene26641 | *PeAQP*_PH02Gene16291 | 0.999393 | 1.79E-05 |
| --- | --- | --- | --- |
| *PeSWEET*_PH02Gene29130 | *PeAQP*_PH02Gene33633 | 0.991406 | 0.000955 |
| *PeSWEET*_PH02Gene23481 | *PeSTP*_PH02Gene37329 | 0.982541 | 0.002762 |
| Fructose content | *PeNINV8* | 0.981956 | 0.002902 |
| *PeSWEET*_PH02Gene29130 | *PeNINV8* | 0.973563 | 0.005140 |
| Fructose content | *PeSWEET*_PH02Gene29130 | 0.972484 | 0.005457 |
| Fructose content | *PeAQP*_PH02Gene33633 | 0.969520 | 0.006358 |
| Glucose content | *PePLT*_PH02Gene17909 | 0.967803 | 0.006902 |
| Glucose content | Fructose content | 0.967502 | 0.006998 |
| Glucose content | *PeAQP*_PH02Gene33633 | 0.957171 | 0.010571 |
| Sucrose content | *PeAQP*_PH02Gene34465 | 0.951521 | 0.012720 |
| *PeAQP*_PH02Gene33633 | *PeNINV8* | 0.948335 | 0.013987 |
| *PeAQP*_PH02Gene34465 | *PeNINV14* | 0.943831 | 0.015845 |
| *PeAQP*_PH02Gene16291 | *PeNINV8* | 0.938965 | 0.017934 |
| *PePLT*_PH02Gene17909 | *PeAQP*_PH02Gene33633 | 0.933247 | 0.020495 |
| *PeAQP*_PH02Gene26641 | *PeNINV8* | 0.932230 | 0.020962 |
| *PeVINV2* | *PeSTP*_PH02Gene37329 | 0.931187 | 0.021444 |
| Glucose content | *PeSWEET*_PH02Gene29130 | 0.927727 | 0.023069 |
| *PeAQP*_PH02Gene34465 | *PeCWINV8* | 0.919414 | 0.027127 |
| Sucrose content | *PeCWINV8* | 0.917226 | 0.028230 |
| Glucose content | *PeNINV8* | 0.911202 | 0.031338 |
| *PeVINV2* | *PeSWEET*_PH02Gene23481 | 0.910160 | 0.031886 |
| Fructose content | *PePLT*_PH02Gene17909 | 0.909474 | 0.032249 |
| Sucrose content | *PeNINV14* | 0.906130 | 0.034034 |
| Fructose content | *PeAQP*_PH02Gene16291 | 0.905621 | 0.034309 |
| Fructose content | *PeAQP*_PH02Gene26641 | 0.899823 | 0.037484 |
| *PeAQP*_PH02Gene34465 | *PePLT*_PH02Gene17909 | 0.89399 | 0.040768 |
| *PePLT*_PH02Gene17908 | *PeNINV14* | 0.893245 | 0.041194 |
| *PePLT*_PH02Gene17909 | *PeCWINV8* | 0.885887 | 0.045474 |
| *PeSWEET*_PH02Gene29130 | *PePLT*_PH02Gene17909 | 0.885048 | 0.045970 |
| *PeSWEET*_PH02Gene29130 | *PeAQP*_PH02Gene16291 | 0.839519 | 0.075288 |
| *PeAQP*_PH02Gene34465 | *PeAQP*_PH02Gene33633 | 0.834523 | 0.078769 |
| *PeAQP*_PH02Gene26641 | *PeSWEET*_PH02Gene29130 | 0.827848 | 0.083494 |
| Glucose content | *PeAQP*_PH02Gene16291 | 0.825889 | 0.084897 |
| *PePLT*_PH02Gene17909 | *PeNINV8* | 0.822274 | 0.087505 |
| Sucrose content | *PePLT*_PH02Gene17908 | 0.818206 | 0.090467 |
| Glucose content | *PeAQP*_PH02Gene26641 | 0.817414 | 0.091047 |
| *PeAQP*_PH02Gene34465 | *PePLT*_PH02Gene17908 | 0.816035 | 0.092060 |
| *PeAQP*_PH02Gene33633 | *PeNINV14* | 0.810220 | 0.096370 |
