# Supplementary material for: Identification and Expression Analyses of Invertase Genes in Moso Bamboo Reveal Their Potential Drought Stress Functions
Source: Front Genet. 2021 Aug 30;12:696300. doi: 10.3389/fgene.2021.696300 (PMC8435750; doi:10.3389/fgene.2021.696300)
Supplement: Supplementary file 1 [file Data_Sheet_1.zip › Supplementary Material/Supplementary Table 3.docx]

**Supplementary Table 3. Putative basic physical and chemical characteristics of PeINVs, BaINVs and OlINVs.**

| Amino acid name | Gene assembly name | Protein size (aa) | Molecular weight (kDa) | Theoretical isoelectric point | Instability index | Ortholog in rice | Ortholog in Arabidopsis |
| --- | --- | --- | --- | --- | --- | --- | --- |
| PeNINV1 | PH02Gene05233.t1 | 475 | 53.04362 | 5.35 | 45.62 | OsNINV2 | AtNINV5 |
| PeNINV2 | PH02Gene05714.t2 | 604 | 67.80156 | 6.35 | 45.07 | OsNINV5 | AtNINV6 |
| PeNINV3 | PH02Gene06169.t1 | 503 | 55.65831 | 5.90 | 54.11 | OsNINV6 | AtNINV6 |
| PeNINV4 | PH02Gene06174.t1 | 606 | 67.35523 | 6.66 | 50.47 | OsNINV6 | AtNINV6 |
| PeNINV5 | PH02Gene08042.t1 | 620 | 69.39640 | 5.53 | 51.72 | OsNINV7 | AtNINV9 |
| PeNINV6 | PH02Gene10235.t1 | 566 | 64.35201 | 6.38 | 48.89 | OsNINV4 | AtNINV4 |
| PeNINV7 | PH02Gene10514.t1 | 549 | 62.84926 | 6.26 | 57.21 | OsNINV3 | AtNINV3 |
| PeNINV8 | PH02Gene19574.t1 | 620 | 69.40745 | 5.66 | 50.48 | OsNINV7 | AtNINV9 |
| PeNINV9 | PH02Gene19699.t1 | 620 | 69.44722 | 5.66 | 52.10 | OsNINV8 | AtNINV9 |
| PeNINV10 | PH02Gene21855.t1 | 497 | 55.52679 | 9.39 | 44.44 | OsNINV6 | AtNINV6 |
| PeNINV11 | PH02Gene27967.t1 | 572 | 63.90046 | 6.22 | 45.00 | OsNINV2 | AtNINV5 |
| PeNINV12 | PH02Gene27972.t1 | 410 | 46.77447 | 6.63 | 43.37 | OsNINV2 | AtNINV5 |
| PeNINV13 | PH02Gene30661.t1 | 559 | 63.01851 | 6.30 | 48.10 | OsNINV1 | AtNINV5 |
| PeNINV14 | PH02Gene31027.t1 | 561 | 63.19153 | 6.13 | 46.89 | OsNINV1 | AtNINV5 |
| PeNINV15 | PH02Gene34390.t1 | 555 | 63.39773 | 6.31 | 50.88 | OsNINV1 | AtNINV5 |
| PeCWINV1 | PH02Gene00392.t1 | 590 | 64.37626 | 5.11 | 40.19 | OsCWINV7 | AtCWINV6 |
| PeCWINV2 | PH02Gene06193.t1 | 584 | 65.40538 | 9.07 | 39.09 | OsCWINV3 | AtCWINV4 |
| PeCWINV3 | PH02Gene06876.t1 | 583 | 66.00063 | 6.09 | 41.15 | OsCWINV5 | AtCWINV6 |
| PeCWINV4 | PH02Gene16851.t1 | 580 | 65.55743 | 8.05 | 29.73 | OsCWINV2 | AtCWINV5 |
| PeCWINV5 | PH02Gene16852.t1 | 582 | 64.94969 | 9.10 | 38.04 | OsCWINV3 | AtCWINV5 |
| PeCWINV6 | PH02Gene20563.t1 | 558 | 61.71981 | 5.37 | 34.56 | OsCWINV9 | AtCWINV6 |
| PeCWINV7 | PH02Gene20565.t1 | 558 | 61.71981 | 5.37 | 34.56 | OsCWINV9 | AtCWINV6 |
| PeCWINV8 | PH02Gene26446.t1 | 576 | 64.30424 | 8.72 | 28.22 | OsCWINV3 | AtCWINV5 |
| PeCWINV9 | PH02Gene27247.t1 | 577 | 64.43565 | 5.79 | 31.80 | OsCWINV2 | AtCWINV5 |
| PeCWINV10 | PH02Gene30701.t1 | 576 | 64.78357 | 6.58 | 34.31 | OsCWINV3 | AtCWINV5 |
| PeVINV1 | PH02Gene02057.t1 | 658 | 72.12241 | 5.32 | 34.34 | OsVINV2 | AtVINV1 |
| PeVINV2 | PH02Gene05689.t1 | 662 | 72.19208 | 5.62 | 35.36 | OsVINV1 | AtVINV2 |
| PeVINV3 | PH02Gene09580.t1 | 671 | 73.69271 | 5.30 | 38.86 | OsVINV1 | AtVINV2 |
| PeVINV4 | PH02Gene09732.t1 | 653 | 71.71486 | 5.17 | 38.88 | OsVINV2 | AtVINV1 |
| BaNINV1 | Bam001043.1 | 432 | 49.36965 | 5.63 | 47.60 | OsNINV7 | AtNINV8 |
| BaNINV2 | Bam005147.1 | 582 | 65.28975 | 6.58 | 43.40 | OsNINV5 | AtNINV6 |
| BaNINV3 | Bam006808.1 | 562 | 63.19061 | 6.30 | 42.74 | OsNINV2. | AtNINV4 |
| BaNINV4 | Bam007340.1 | 549 | 62.67894 | 6.10 | 55.26 | OsNINV3 | AtNINV5 |
| BaNINV5 | Bam007666.1 | 465 | 53.17541 | 6.80 | 49.86 | OsNINV4 | AtNINV5 |
| BaNINV6 | Bam015155.1 | 622 | 69.53336 | 5.55 | 54.93 | OsNINV8 | AtNINV7 |
| BaNINV7 | Bam024009.1 | 614 | 68.66634 | 5.58 | 52.41 | OsNINV7 | AtNINV8 |
| BaNINV8 | Bam025610.1 | 398 | 44.37133 | 5.80 | 51.09 | OsNINV8 | AtNINV7 |
| BaNINV9 | Bam025666.1 | 582 | 65.36886 | 6.36 | 45.38 | OsNINV5 | AtNINV6 |
| BaNINV10 | Bam026459.1 | 500 | 55.62522 | 5.13 | 49.32 | OsNINV8 | AtNINV7 |
| BaNINV11 | Bam029287.1 | 562 | 63.34788 | 6.30 | 49.00 | OsNINV1 | AtNINV4 |
| BaNINV12 | Bam030011.1 | 466 | 53.16450 | 8.48 | 55.18 | OsNINV3 | AtNINV5 |
| BaNINV13 | Bam034196.1 | 564 | 63.37995 | 6.42 | 48.19 | OsNINV2 | AtNINV4 |
| BaNINV14 | Bam038656.1 | 549 | 62.69297 | 6.10 | 55.56 | OsNINV3 | AtNINV5 |
| BaNINV15 | Bam046364.1 | 561 | 63.05138 | 6.52 | 45.34 | OsNINV2 | AtNINV4 |
| BaNINV16 | Bam046618.1 | 550 | 62.94855 | 6.26 | 55.37 | OsNINV3 | AtNINV5 |
| BaCWINV1 | Bam003797.1 | 434 | 48.72056 | 5.94 | 36.83 | OsCWINV7 | AtCWINV1 |
| BaCWINV2 | Bam007033.1 | 693 | 77.06056 | 6.91 | 30.71 | OsCWINV4 | AtCWINV5 |
| BaCWINV3 | Bam021914.3 | 524 | 58.07534 | 5.98 | 38.61 | OsCWINV8 | AtCWINV1 |
| BaCWINV4 | Bam032618.1 | 577 | 64.40611 | 8.88 | 34.36 | OsCWINV3 | AtCWINV5 |
| BaCWINV5 | Bam032619.1 | 522 | 58.65138 | 8.68 | 34.32 | OsCWINV3 | AtCWINV5 |
| BaCWINV6 | Bam035627.1 | 573 | 64.08896 | 8.32 | 29.56 | OsCWINV4 | AtCWINV5 |
| BaCWINV7 | Bam039826.1 | 412 | 46.75805 | 8.91 | 43.16 | OsCWINV3 | AtCWINV5 |
| BaCWINV8 | Bam041578.1 | 573 | 64.26507 | 8.07 | 30.15 | OsCWINV4 | AtCWINV5 |
| BaVINV1 | Bam003971.1 | 659 | 72.07111 | 5.05 | 40.31 | OsVINV2 | AtVINV1 |
| BaVINV2 | Bam004818.1 | 656 | 71.93669 | 5.71 | 36.30 | OsVINV1 | AtVINV2 |
| BaVINV3 | Bam008116.1 | 590 | 64.66971 | 5.75 | 34.35 | OsVINV1 | AtVINV2 |
| BaVINV4 | Bam034484.1 | 645 | 70.44639 | 5.21 | 37.59 | OsVINV2 | AtVINV1 |
| OlNINV1 | Ola007832.1 | 604 | 67.84767 | 6.90 | 47.12 | OsNINV5 | AtNINV6 |
| OlNINV2 | Ola013911.1 | 612 | 68.38007 | 5.66 | 50.77 | OsNINV7 | AtNINV8 |
| OlNINV3 | Ola018025.1 | 565 | 63.89864 | 6.92 | 50.13 | OsNINV4 | AtNINV5 |
| OlNINV4 | Ola018029.1 | 545 | 61.77625 | 7.55 | 51.09 | OsNINV4 | AtNINV5 |
| OlNINV5 | Ola019342.1 | 572 | 63.97473 | 6.41 | 44.06 | OsNINV2 | AtNINV4 |
| OlNINV6 | Ola020740.1 | 614 | 68.67928 | 6.12 | 50.85 | OsNINV8 | AtNINV7 |
| OlNINV7 | Ola031357.1 | 549 | 62.74512 | 5.91 | 55.91 | OsNINV3 | AtNINV5 |
| OlNINV8 | Ola039050.2 | 504 | 56.58694 | 6.21 | 49.08 | OsNINV1 | AtNINV4 |
| OlCWINV1 | Ola000897.1 | 583 | 64.54179 | 5.96 | 43.49 | OsCWINV8 | AtCWINV1 |
| OlCWINV2 | Ola004900.1 | 667 | 75.22971 | 8.89 | 42.24 | OsCWINV5 | AtCWINV1 |
| OlCWINV3 | Ola005595.1 | 587 | 65.38957 | 9.33 | 37.41 | OsCWINV3 | AtCWINV5 |
| OlCWINV4 | Ola005596.1 | 582 | 65.69960 | 8.57 | 28.30 | OsCWINV2 | AtCWINV5 |
| OlCWINV5 | Ola007632.1 | 577 | 64.23247 | 5.83 | 32.08 | OsCWINV1 | AtCWINV5 |
| OlCWINV6 | Ola007783.1 | 558 | 62.91776 | 8.88 | 34.57 | OsCWINV4 | AtCWINV5 |
| OlCWINV7 | Ola033474.1 | 556 | 60.93506 | 5.22 | 32.41 | OsCWINV7 | AtCWINV1 |
| OlVINV1 | Ola025141.1 | 462 | 50.32879 | 5.58 | 43.81 | OsVINV2 | AtVINV2 |
| OlVINV2 | Ola031057.1 | 646 | 70.52945 | 5.96 | 40.39 | OsVINV1 | AtVINV1 |
