# Supplementary material for: Identification and Expression Analyses of Invertase Genes in Moso Bamboo Reveal Their Potential Drought Stress Functions
Source: Front Genet. 2021 Aug 30;12:696300. doi: 10.3389/fgene.2021.696300 (PMC8435750; doi:10.3389/fgene.2021.696300)
Supplement: Supplementary file 1 [file Data_Sheet_1.zip › Supplementary Material/Supplementary Table 5.docx]

**Supplementary Table 5. The nonsynonymous substitution (*Ka*), synonymous substitution (*Ks*), and *Ka*/*Ks* ratios of homologous gene pair of *INV*s.**

| Gene_1 | Gene_2 | Nonsynonymous substitution (*Ka*) | Synonymous substitution (*Ks*) | Nonsynonymous/synonymous (*Ka*/*Ks*) |
| --- | --- | --- | --- | --- |
| *PeCWINV8* | *PeCWINV2* | 0.395712 | 0.946567 | 0.418049 |
| *PeNINV1* | *PeNINV11* | 0.025838 | 0.128785 | 0.200631 |
| *PeNINV1* | *PeNINV13* | 0.114301 | 1.359221 | 0.084093 |
| *PeNINV14* | *PeNINV1* | 0.107054 | 1.484898 | 0.072095 |
| *PeNINV14* | *PeNINV12* | 0.077594 | 2.764980 | 0.028063 |
| *PeNINV14* | *PeNINV13* | 0.018971 | 0.088377 | 0.214659 |
| *PeNINV4* | *PeNINV2* | 0.141574 | 0.902864 | 0.156806 |
| *PeNINV5* | *PeNINV8* | 0.018282 | 0.165373 | 0.110548 |
| *PeVINV4* | *PeVINV1* | 0.029904 | 0.102180 | 0.292656 |
| *PeCWINV1* | *OsCWINV7* | 0.108234 | 0.434897 | 0.248872 |
| *PeCWINV2* | *OsCWINV4* | 0.160807 | 0.821395 | 0.195774 |
| *PeCWINV3* | *OsCWINV5* | 0.053158 | 0.506503 | 0.104952 |
| *PeCWINV5* | *OsCWINV4* | 0.155310 | 0.907127 | 0.171210 |
| *PeCWINV8* | *OsCWINV4* | 0.414972 | 1.059448 | 0.391687 |
| *PeCWINV8* | *OsCWINV2* | 0.421126 | 0.967488 | 0.435278 |
| *PeCWINV9* | *OsCWINV1* | 0.450318 | 1.107054 | 0.406772 |
| *PeNINV1* | *OsNINV2* | 0.061643 | 0.458509 | 0.134442 |
| *PeNINV11* | *OsNINV1* | 0.092543 | 1.169083 | 0.079159 |
| *PeNINV12* | *OsNINV1* | 0.073023 | 2.768481 | 0.026377 |
| *PeNINV12* | *OsNINV2* | 0.060089 | 0.694746 | 0.086491 |
| *PeNINV14* | *OsNINV1* | 0.025902 | 0.501896 | 0.051608 |
| *PeNINV14* | *OsNINV2* | 0.118173 | 1.673964 | 0.070594 |
| *PeNINV2* | *OsNINV5* | 0.040305 | 0.401257 | 0.100448 |
| *PeNINV2* | *OsNINV6* | 0.122374 | 1.059152 | 0.115539 |
| *PeNINV4* | *OsNINV5* | 0.157639 | 1.083104 | 0.145544 |
| *PeNINV4* | *OsNINV6* | 0.084665 | 0.338463 | 0.250145 |
| *PeNINV5* | *OsNINV7* | 0.041775 | 0.371943 | 0.112316 |
| *PeNINV6* | *OsNINV4* | 0.067010 | 0.475307 | 0.140982 |
| *PeNINV7* | *OsNINV3* | 0.019281 | 0.564554 | 0.034152 |
| *PeNINV8* | *OsNINV7* | 0.040205 | 0.359850 | 0.111726 |
| *PeNINV9* | *OsNINV8* | 0.055192 | 0.345884 | 0.159569 |
| *PeVINV1* | *OsVINV2* | 0.083222 | 0.344183 | 0.241797 |
| *PeVINV2* | *OsVINV1* | 0.117871 | 0.325324 | 0.362319 |
| *PeVINV4* | *OsVINV2* | 0.081877 | 0.344736 | 0.237506 |
| *PeVINV1* | *BaCWINV1* | 0.036407 | 0.132642 | 0.274475 |
| *PeVINV4* | *BaCWINV1* | 0.035624 | 0.139605 | 0.255180 |
| *PeCWINV1* | *BaCWINV3* | 0.137143 | 0.421950 | 0.325022 |
| *PeCWINV2* | *BaCWINV4* | 0.039649 | 0.239107 | 0.165822 |
| *PeCWINV2* | *BaCWINV6* | 0.145820 | 0.726915 | 0.200601 |
| *PeCWINV8* | *BaCWINV6* | 0.037441 | 0.225534 | 0.166012 |
| *PeCWINV2* | *BaCWINV7* | 0.029138 | 0.157761 | 0.184697 |
| *PeCWINV8* | *BaCWINV7* | 0.181759 | 0.642128 | 0.283056 |
| *PeCWINV8* | *BaCWINV8* | 0.032582 | 0.197567 | 0.164915 |
| *PeNINV9* | *BaNINV10* | 0.020666 | 0.107433 | 0.192364 |
| *PeNINV1* | *BaNINV13* | 0.107479 | 1.340250 | 0.080193 |
| *PeNINV12* | *BaNINV13* | 0.079128 | 2.215658 | 0.035713 |
| *PeNINV13* | *BaNINV13* | 0.019579 | 0.063826 | 0.306758 |
| *PeNINV14* | *BaNINV13* | 0.013150 | 0.105733 | 0.124367 |
| *PeNINV1* | *BaNINV15* | 0.021031 | 0.135308 | 0.155427 |
| *PeNINV12* | *BaNINV15* | 0.016660 | 0.154982 | 0.107496 |
| *PeNINV13* | *BaNINV16* | 0.157545 | - | - |
| *PeNINV14* | *BaNINV16* | 0.154636 | - | - |
| *PeNINV7* | *BaNINV16* | 0.013950 | 0.191964 | 0.072671 |
| *PeNINV1* | *BaNINV3* | 0.028145 | 0.116676 | 0.241219 |
| *PeNINV12* | *BaNINV3* | 0.017233 | 0.160406 | 0.107433 |
| *PeNINV14* | *BaNINV3* | 0.086847 | 1.348435 | 0.064406 |
| *PeNINV7* | *BaNINV4* | 0.011946 | 0.169985 | 0.070278 |
| *PeNINV6* | *BaNINV5* | 0.016899 | 0.083095 | 0.203374 |
| *PeNINV5* | *BaNINV7* | 0.017979 | 0.149399 | 0.120345 |
| *PeNINV8* | *BaNINV7* | 0.017387 | 0.145687 | 0.119344 |
| *PeVINV2* | *BaVINV3* | 0.046890 | 0.171309 | 0.273717 |
| *PeVINV1* | *BaVINV4* | 0.036615 | 0.131300 | 0.278862 |
| *PeVINV4* | *BaVINV4* | 0.041350 | 0.143531 | 0.288091 |
| *PeCWINV1* | *OlCWINV1* | 0.168451 | 0.471692 | 0.357120 |
| *PeCWINV2* | *OlCWINV3* | 0.089698 | 0.366902 | 0.244473 |
| *PeCWINV8* | *OlCWINV3* | 0.193408 | 0.699361 | 0.276550 |
| *PeCWINV9* | *OlCWINV5* | 0.072096 | 0.250265 | 0.288078 |
| *PeNINV2* | *OlCWINV5* | 0.023034 | 0.182269 | 0.126372 |
| *PeNINV4* | *OlCWINV5* | 0.130621 | 1.047232 | 0.124730 |
| *PeNINV5* | *OlNINV2* | 0.017444 | 0.227058 | 0.076825 |
| *PeNINV8* | *OlNINV2* | 0.017098 | 0.23058 | 0.074152 |
| *PeNINV6* | *OlNINV3* | 0.057794 | 0.258058 | 0.223956 |
| *PeNINV6* | *OlNINV4* | 0.052664 | 0.217146 | 0.242528 |
| *PeNINV1* | *OlNINV5* | 0.033589 | 0.227288 | 0.147780 |
| *PeNINV12* | *OlNINV5* | 0.029917 | 0.337768 | 0.088572 |
| *PeNINV14* | *OlNINV5* | 0.099220 | 1.627832 | 0.060952 |
| *PeNINV9* | *OlNINV6* | 0.031466 | 0.181426 | 0.173437 |
| *PeNINV1* | *OlNINV8* | 0.114738 | 1.233199 | 0.093041 |
| *PeNINV12* | *OlNINV8* | 0.077010 | 2.504902 | 0.030744 |
| *PeNINV13* | *OlNINV8* | 0.020274 | 0.224114 | 0.090465 |
| *PeNINV14* | *OlNINV8* | 0.009597 | 0.237642 | 0.040383 |
| *PeVINV2* | *OlVINV2* | 0.079463 | 0.286222 | 0.277626 |
| *BaVINV2* | *OlVINV2* | 0.083677 | 0.326077 | 0.256618 |
| *BaNINV3* | *OlNINV5* | 0.033691 | 0.261102 | 0.129034 |
| *BaNINV3* | *OlNINV8* | 0.088006 | 1.238906 | 0.071035 |
| *BaNINV5* | *OlNINV4* | 0.042026 | 0.246956 | 0.170175 |
| *BaVINV3* | *OlVINV2* | 0.086040 | 0.328926 | 0.261579 |
| *BaNINV8* | *OlNINV6* | 0.074815 | 0.272343 | 0.274709 |
| *BaNINV13* | *OlNINV8* | 0.010474 | 0.218791 | 0.047874 |
| *BaCWINV3* | *OlCWINV1* | 0.060941 | 0.189834 | 0.321021 |
| *BaCWINV7* | *OlCWINV3* | 0.060519 | 0.337110 | 0.179523 |
| *BaCWINV8* | *OlCWINV3* | 0.184673 | 0.784244 | 0.235479 |
| *BaNINV7* | *OlNINV2* | 0.013420 | 0.218904 | 0.061305 |
| *BaNINV15* | *OlNINV5* | 0.027561 | 0.249114 | 0.110635 |
