# Supplementary material for: Identification and Expression Analyses of Invertase Genes in Moso Bamboo Reveal Their Potential Drought Stress Functions
Source: Front Genet. 2021 Aug 30;12:696300. doi: 10.3389/fgene.2021.696300 (PMC8435750; doi:10.3389/fgene.2021.696300)
Supplement: Supplementary file 1 [file Data_Sheet_1.zip › Supplementary Material/Supplementary Table 6.docx]

**Supplementary Table 6. The Pearson’s correlation coefficients (PCC) of** **sugar contents and the gene expression levels of *PeINVs* and *SWTGs* under drought stress.**

| Group_A | Group_B | Correlation coefficients | *P*-values |
| --- | --- | --- | --- |

| *PeAQP*_PH02Gene26641 | *PeAQP*_PH02Gene16291 | 0.999393 | 1.79E-05 |
| --- | --- | --- | --- |
| *PeSWEET*_PH02Gene29130 | *PeAQP*_PH02Gene33633 | 0.991406 | 0.000955 |
| *PeSWEET*_PH02Gene23481 | *PeSTP*_PH02Gene37329 | 0.982541 | 0.002762 |
| Fructose content | *PeNINV8* | 0.981956 | 0.002902 |
| *PeSWEET*_PH02Gene29130 | *PeNINV8* | 0.973563 | 0.005140 |
| Fructose content | *PeSWEET*_PH02Gene29130 | 0.972484 | 0.005457 |
| Fructose content | *PeAQP*_PH02Gene33633 | 0.969520 | 0.006358 |
| Glucose content | *PePLT*_PH02Gene17909 | 0.967803 | 0.006902 |
| Glucose content | Fructose content | 0.967502 | 0.006998 |
| Glucose content | *PeAQP*_PH02Gene33633 | 0.957171 | 0.010571 |
| Sucrose content | *PeAQP*_PH02Gene34465 | 0.951521 | 0.012720 |
| *PeAQP*_PH02Gene33633 | *PeNINV8* | 0.948335 | 0.013987 |
| *PeAQP*_PH02Gene34465 | *PeNINV14* | 0.943831 | 0.015845 |
| *PeAQP*_PH02Gene16291 | *PeNINV8* | 0.938965 | 0.017934 |
| *PePLT*_PH02Gene17909 | *PeAQP*_PH02Gene33633 | 0.933247 | 0.020495 |
| *PeAQP*_PH02Gene26641 | *PeNINV8* | 0.932230 | 0.020962 |
| *PeVINV2* | *PeSTP*_PH02Gene37329 | 0.931187 | 0.021444 |
| Glucose content | *PeSWEET*_PH02Gene29130 | 0.927727 | 0.023069 |
| *PeAQP*_PH02Gene34465 | *PeCWINV8* | 0.919414 | 0.027127 |
| Sucrose content | *PeCWINV8* | 0.917226 | 0.028230 |
| Glucose content | *PeNINV8* | 0.911202 | 0.031338 |
| *PeVINV2* | *PeSWEET*_PH02Gene23481 | 0.910160 | 0.031886 |
| Fructose content | *PePLT*_PH02Gene17909 | 0.909474 | 0.032249 |
| Sucrose content | *PeNINV14* | 0.906130 | 0.034034 |
| Fructose content | *PeAQP*_PH02Gene16291 | 0.905621 | 0.034309 |
| Fructose content | *PeAQP*_PH02Gene26641 | 0.899823 | 0.037484 |
| *PeAQP*_PH02Gene34465 | *PePLT*_PH02Gene17909 | 0.89399 | 0.040768 |
| *PePLT*_PH02Gene17908 | *PeNINV14* | 0.893245 | 0.041194 |
| *PePLT*_PH02Gene17909 | *PeCWINV8* | 0.885887 | 0.045474 |
| *PeSWEET*_PH02Gene29130 | *PePLT*_PH02Gene17909 | 0.885048 | 0.045970 |
| *PeSWEET*_PH02Gene29130 | *PeAQP*_PH02Gene16291 | 0.839519 | 0.075288 |
| *PeAQP*_PH02Gene34465 | *PeAQP*_PH02Gene33633 | 0.834523 | 0.078769 |
| *PeAQP*_PH02Gene26641 | *PeSWEET*_PH02Gene29130 | 0.827848 | 0.083494 |
| Glucose content | *PeAQP*_PH02Gene16291 | 0.825889 | 0.084897 |
| *PePLT*_PH02Gene17909 | *PeNINV8* | 0.822274 | 0.087505 |
| Sucrose content | *PePLT*_PH02Gene17908 | 0.818206 | 0.090467 |
| Glucose content | *PeAQP*_PH02Gene26641 | 0.817414 | 0.091047 |
| *PeAQP*_PH02Gene34465 | *PePLT*_PH02Gene17908 | 0.816035 | 0.092060 |
| *PeAQP*_PH02Gene33633 | *PeNINV14* | 0.810220 | 0.096370 |
